# C-terminal frameshift variant of TDP-43 with pronounced aggregation-propensity causes rimmed vacuole myopathy but not ALS/FTD

**DOI:** 10.1007/s00401-023-02565-1

**Published:** 2023-03-31

**Authors:** Pedro Ervilha Pereira, Nika Schuermans, Antoon Meylemans, Pontus LeBlanc, Lauren Versluys, Katie E. Copley, Jack D. Rubien, Christopher Altheimer, Myra Peetermans, Elke Debackere, Olivier Vanakker, Sandra Janssens, Jonathan Baets, Kristof Verhoeven, Martin Lammens, Sofie Symoens, Boel De Paepe, Sami J. Barmada, James Shorter, Jan L. De Bleecker, Elke Bogaert, Bart Dermaut

**Affiliations:** 1 Center for Medical Genetics, Ghent University Hospital, Ghent, Belgium; 2 Department of Biomolecular Medicine, Faculty of Medicine and Health Sciences, Ghent University, Ghent, Belgium; 3 Department of Neurology, Ghent University Hospital, Ghent, Belgium; 4 Department of Head and Skin, Faculty of Medicine and Health Sciences, Ghent University, Ghent, Belgium; 5 Department of Biochemistry and Biophysics, Perelman School of Medicine, University of Pennsylvania, Philadelphia, PA 19104, USA; 6 Neuroscience Graduate Group, Perelman School of Medicine, University of Pennsylvania, Philadelphia, PA 19104, USA; 7 Department of Neurology, University of Michigan, Ann Arbor, MI 48109, USA; 8 Department of Neurology, Neuromuscular Reference Centre, Antwerp University Hospital, Antwerp, Belgium; 9 Faculty of Medicine and Health Sciences, Translational Neurosciences, University of Antwerp, Antwerp, Belgium; 10 Laboratory of Neuromuscular Pathology, Institute Born-Bunge, University of Antwerp, Antwerp, Belgium; 11 Department of Neurology, Sint-Jan Hospital Bruges, Brugge, Belgium; 12 Department of Pathology, Antwerp University Hospital, University of Antwerp, Edegem, Belgium

**Keywords:** TDP-43, Myopathy, ALS/FTD, Genetics, *Drosophila*, Phase separation

## Abstract

Neuronal TDP-43-positive inclusions are neuropathological hallmark lesions in frontotemporal dementia (FTD) and amyotrophic lateral sclerosis (ALS). Pathogenic missense variants in *TARDBP*, the gene encoding TDP-43, can cause ALS and cluster in the C-terminal prion-like domain (PrLD), where they modulate the liquid condensation and aggregation properties of the protein. TDP-43-positive inclusions are also found in rimmed vacuole myopathies, including sporadic inclusion body myositis, but myopathy-causing TDP-43 variants have not been reported. Using genome-wide linkage analysis and whole exome sequencing in an extended five-generation family with an autosomal dominant rimmed vacuole myopathy, we identified a conclusively linked frameshift mutation in TDP-43 producing a C-terminally altered PrLD (TDP-43^p.Trp385IlefsTer10^) (maximum multipoint LOD-score 3.61). Patient-derived muscle biopsies showed TDP-43-positive sarcoplasmic inclusions, accumulation of autophagosomes and transcriptomes with abnormally spliced sarcomeric genes (including *TTN* and *NEB*) and increased expression of muscle regeneration genes. In vitro phase separation assays demonstrated that TDP-43^Trp385IlefsTer10^ does not form liquid-like condensates and readily forms solid-like fibrils indicating increased aggregation propensity compared to wild-type TDP-43. In *Drosophila* TDP-43^p.Trp385IlefsTer10^ behaved as a partial loss-of-function allele as it was able to rescue the *TBPH* (fly ortholog of *TARDBP*) neurodevelopmental lethal null phenotype while showing strongly reduced toxic gain-of-function properties upon overexpression. Accordingly, TDP-43^p.Trp385IlefsTer10^ showed reduced toxicity in a primary rat neuron disease model. Together, these genetic, pathological, in vitro and in vivo results demonstrate that TDP-43^p.Trp385IlefsTer10^ is an aggregation-prone partial loss-of-function variant that causes autosomal dominant vacuolar myopathy but not ALS/FTD. Our study genetically links TDP-43 proteinopathy to myodegeneration, and reveals a tissue-specific role of the PrLD in directing pathology.

## Introduction

TDP-43, encoded by *TARDBP*, is an evolutionary highly conserved and ubiquitously expressed RNA- and DNA-binding protein (reviewed in [57]). It is involved in multiple pathways of RNA metabolism, including pre-mRNA splicing, RNA transport, mRNA stability, miRNA processing and stress granule assembly. It is predominantly localized in the nucleus but also shuttles to the cytoplasm. The protein contains a nuclear localization signal (NLS) within its N-terminal domain (NTD), two RNA recognition motifs (RMM1, RMM2) and a C-terminal region (aa.272-414), which is highly disordered and forms a low complexity domain (LCD) that is similar to that of prion-like domains (PrLD) of yeast proteins [23, 44]. The PrLD of TDP-43 is responsible for protein–protein interactions and is needed for the proper functioning of the protein through the process of liquid-liquid phase separation (LLPS) [21].

Nearly all cases of amyotrophic lateral sclerosis (ALS) and ~ 45% of frontotemporal dementia (FTD) patients are neuropathologically characterized by neuronal cytoplasmic TDP-43 inclusions accompanied by a nuclear depletion of TDP-43 [1, 42]. Pathogenic missense variants in *TARDBP* [15, 26, 53], which cluster in the PrLD, account for ~ 4% of familial ALS cases and < 1% of sporadic ALS cases [27]. These findings indicate that the PrLD of TDP-43 is crucially involved in ALS/FTD pathogenesis and that altered LLPS-related physical-chemical properties of this region, which are essential for the formation of TDP-43-associated stress granules and RNA binding, could lead to the irreversible formation of solid aggregates [21, 57]. Interestingly, a cryo-electron microscopy study of TDP-43 filaments from brain cortices of ALS/FTD patients revealed an amyloid-like structure of which the filament core spans aa. 282-360 in the PrLD and adopts a double-spiral-shaped fold [4]. Although such filaments are generally assumed to be neurotoxic [32], the exact pathogenic roles of insoluble aggregates and liquid-like condensates are still unclear and under debate [25]. Based on an unbiased deep mutagenesis study in yeast, Bolognesi et al. suggested that TDP-43 aggregates are protective, and that cellular toxicity is caused by liquid-like TDP-43 condensates [8]. Therefore, whether TDP-43-mediated neuronal cell death results from a toxic gain-of-function of cytoplasmic TDP-43, as either aggregates or liquid-like droplets, and/or a loss of its normal function due to nuclear depletion remains unknown [11].

Although TDP-43 aggregates are a typical cellular hallmark of neurodegeneration, its loss-of-function has been associated with myodegeneration in zebrafish [50] and *Drosophila* [16, 36]. In humans, accumulation of TDP-43 is also frequently observed in myopathies with rimmed vacuoles [31, 58]. Rimmed vacuoles are a common feature of myopathies in which autophagic impairment plays a crucial pathogenic role [38]. Under the light microscope, rimmed vacuoles appear as empty spaces in the sarcoplasm surrounded by a rim of basophilic granular material. However, their content can be identified in transmission electron microscopy (TEM) preparations as filamentous protein inclusions surrounded by a rim of autophagic material. TDP-43-positive rimmed vacuole myopathies [31] include sporadic inclusion body myositis (sIBM) [10], hereditary inclusion body myopathy with early-onset Paget disease and FTD (caused by heterozygous mutations in *VCP*) [41, 60, 61], inclusion body myopathy associated with multisystem proteinopathy (caused by heterozygous mutations in *HNRNPA2B1* and *HNRNPA1*) [28], distal myopathy with rimmed vacuoles (caused by bi-allelic mutations in *GNE*) [19] and oculopharyngeal muscular dystrophies (caused by heterozygous mutations in *PABPN1, HNRNPA2B1* and *HNRNPA1*) [6, 9, 29]. In addition, Vogler et al. demonstrated that TDP-43 is an essential protein for normal skeletal muscle function, since it is involved in the assembly of sarcoplasmic amyloid-like RNA–protein assemblies called myo-granules, whose presence is increased in damaged tissues with elevated regeneration. Interestingly, myo-granules can seed TDP-43 amyloid-like fibrils in vitro and it has been proposed that the formation of TDP-43 aggregates in the genetically diverse group of rimmed vacuole myopathies is initially triggered by elevated muscle regeneration [59]. Whether TDP-43 aggregation plays a primary role in rimmed vacuole myopathies or is a phenomenon secondary to muscle degeneration is unknown, since genetic evidence linking pathogenic *TARDBP* variants to skeletal muscle disorders has previously been lacking.

We here report a family with multiple affected individuals presenting with an unexplained slowly progressive myopathy with rimmed vacuoles and TDP-43/p62-positive cytoplasmic inclusions caused by a truncating pathogenic variant in the C-terminal PrLD of TDP-43. Further, in silico, in vitro, in vivo, and transcriptomic studies demonstrated that the novel pathogenic variant is aggregation-prone and has partial loss-of-function properties. Our study provides genetic evidence for the crucial role of TDP-43 in the pathogenesis of muscle degeneration.

## Materials and methods

### Patient material and data availability

**Fig. 1:**
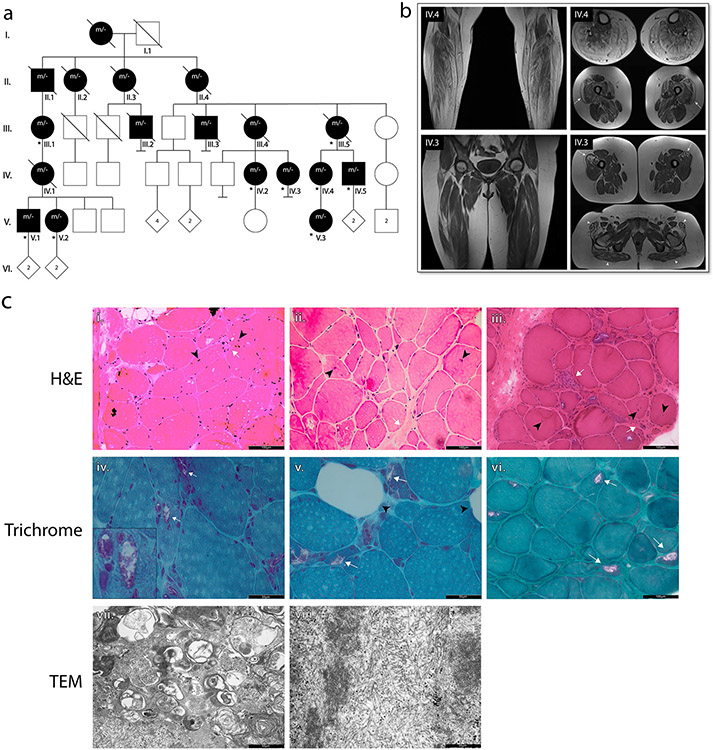
Identification of TDP-43^p.Trp385IlefsTer10^ as a cause of autosomal dominant rimmed vacuole myopathy. **a** Pedigree of the family in which the TDP-43^p.Trp385ilefsTer10^ variant was identified. Individuals identified with “*m/-* “ are affected individuals that have either been genotyped or in which the genotype was reconstructed based on segregation. Individuals that were genotyped are identified with an asterisk (*). **b** Coronal and axial plane T1-weighted MRI images of the lower limb muscles of patients IV.3 and IV.4. Fatty atrophy of the muscles in the anterior, lateral and posterior compartments of the distal lower extremities in patient IV.4 (top left). Global muscular atrophy of the pelvic girdle and proximal lower extremities in patient IV.3 (bottom left). Fatty atrophy of the gluteus maximus and tensor fasciae latae (patient IV.3, bottom right, white arrowheads). Bilateral focal infiltration in the vastus lateralis of the quadriceps muscles (patients IV.3 and IV.4, white arrows). c Histological images of patient muscle biopsies: i-iii. H&E of tissue from patients IV.5, IV.4 and III.5, respectively. Tissue sections show signs of myopathy with an uneven distribution of fibre size, presence of centralized nuclei (black arrowheads) and increased fibrotic tissue proliferation (white arrows). Scale bar = 100 μm. iv-vi. Modified Gomori trichrome staining of muscle tissue from patients IV.5, IV.4 and III.1, respectively. Staining shows the presence of rimmed vacuoles (white arrows), along with infiltration of fatty tissue (black arrowheads). Scale bar = 50 μm. vii. TEM of patient III.5 muscle reveals signs of enhanced autophagy with the presence of multiple autophagic vesicles (black arrow) and viii. the accumulation of fibrous material (black arrowhead)

For this study 2 muscle biopsies were collected from patients (Fig. 1a: IV.4, IV.5). These biopsies were used to generate tissue sections, protein extracts and for RNA extraction. The obtained RNA was used to generate RNA-Seq data. Archived data from 2 additional patients followed in the 1980s (Fig. 1a: III.1, III.5) was available from the pathology department of the University hospital of Antwerp. Original blocks and sections were no longer available for these patients, but histological and TEM images were still available.

### Patient clinical characteristics

All affected individuals, except patients V-1 and V-2, were clinically evaluated by the same neurologist (JDB) with expertise in neuromuscular diseases at the University Hospital of Ghent. Blood samples were obtained for dosage of plasma CK levels. In six patients, an electromyography (EMG) was performed. A muscle MRI was performed in two patients, and in one patient an ultrasound of the lower limb musculature was performed. In four affected family members a muscle biopsy was taken and in one individual a nerve biopsy was performed (Supplementary data, online resource). The study was approved by the Ethics Committee of the University Hospital of Ghent (EC: 2019/1430). Written informed consents for multi-omics analysis and publication of the results were obtained from all patients. Detailed clinical features are described in the Supplementary Data, online resource.

### Genetic studies

Single nucleotide polymorphism (SNP)-array based linkage analysis, whole exome sequencing (WES) and Sanger sequencing of *TARDBP* were performed on genomic DNA extracted from peripheral blood. Genotyping for linkage analysis was performed by Macrogen in ten DNA samples (III.1, III.5, IV.2, IV.3, IV.4, IV.5, V.1, V.2, V.3 and unaffected daughter of IV.4) using 2.3 M genome-wide Infinium Omni2-5-8v1.5 SNP genotyping arrays (Illumina, San Diego, CA). We performed multipoint linkage analysis using the Superlink-Online SNP 1.1 tool [52]. WES in patients III.1 and IV.4 was done on the Illumina HiSeq 3000 Platform after enrichment of gDNA with SureSelectXT Low Input Human All Exon v6 (Agilent Technologies). CLC Genomics Workbench (v9.0.1) software was used for read mapping against the human genome reference sequence (NCBI, GRCh37.p5/hg19). Variant calling and filtering were done using Seqplorer, an in-house developed tool for the analysis of WES-data. The position of the called variants is based on NCBI build GRCh38. A minimum of 90% of the interrogated genes have a coverage of > 20x. Classical Sanger sequencing was performed to check *TARDBP* c.1151-1161del in patients IV.2, IV.3, IV.5, V.1, V.2 and the unaffected daughter of IV.2.

### Muscle biopsy-derived myocyte cultures

Muscle biopsies of the m. quadriceps femoris were obtained from TDP-43^Trp385IlefsTer10^ patients IV.4 and IV.5. Additionally, healthy muscle samples were obtained from the orthopaedic department at the Ghent University Hospital from three patients undergoing hip replacement surgery. Shortly after collection, the tissue was scissor minced, thoroughly washed and incubated in trypsin (0.25%) to further break down the connective tissue and separate the skeletal muscle cells. The resulting fragments and suspended cells were then seeded onto T75 culture flasks and cultured in Dulbecco’s modified Eagle medium (DMEM) supplemented with 20% foetal bovine serum (FBS), penicillin and streptomycin, and kept at 37 °C in a humified atmosphere containing 5% CO_2_. The medium was changed regularly, once a week, until the flasks reached confluency. The cells were then passaged using trypsin (0.25%, Thermofisher Scientific). Expansion of the cell culture was carried out until a number of ± 10 flasks per biopsy was achieved in order to obtain enough cells for successful sorting. To ensure a high myoblast purity, a selection for CD56 + was performed using labelled magnetic beads. To prepare for sorting, 10 flasks of cells per biopsy were trypsinized and the resulting cell suspension was pooled together. The collected cells were labelled with a magnetic bead conjugated anti-CD56 antibody and run through MACS separation columns while attached to a MiniMACS separator magnet. Labelled myogenic progenitor cells were retained while unlabelled cells were eluted. After elution of unlabelled cells, the columns were removed from the magnet and the cells retained eluted as the CD56 + fraction of the original culture. CD56 +cells were seeded onto T75 culture flasks with culture medium (DMEM with 20% FBS and Pen-Strep) and allowed to recover and grow over a few weeks with regular medium changes. Cultures were passaged before reaching confluency and up to a total number of 20 passages.

### Immunofluorescence and TEM

Patient and control muscle biopsies obtained from the m. quadriceps femoris were embedded in optimal cutting temperature (OCT) compound and flash frozen in isopentane. Cryosections were made from these samples and used for immunofluorescent staining. After freezing, 5 μm thick tissue sections were prepared onto SuperFrost^™^ slides and stored at −80 °C until used. To perform immunofluorescence staining the frozen tissue sections were allowed to thaw at room temperature and hydrophobic rings were drawn around each section with a Dako pen (Agilent) to minimize the amount of antibody required for incubation. Tissues were fixed in 4% paraformaldehyde (PFA) for 20 min at −20 °C. Following fixation, the sections were incubated with blocking buffer (1% donkey serum in PBS) and allowed to block for 30 min at room temperature. After blocking, the tissue samples were incubated with primary antibody at the appropriate dilution in blocking buffer at 4 °C, overnight. Prior to secondary incubation, the sections were washed thoroughly with PBS. Secondary antibodies were diluted in blocking solution and used to incubate the tissue samples for 1 h at room temperature. During secondary incubation, samples were kept in the dark. To finalize the staining procedure, the tissue samples were washed thoroughly with PBS and mounted with VECTASHIELD HardSet^™^ mounting medium with DAPI counterstain (Vector Laboratories).

Purified myocyte cultures obtained from the muscle biopsies collected were cultured on glass coverslips and fixed in glutaraldehyde. These samples were embedded in resin, sectioned and used for TEM. Cells were seeded on glass coverslips and allowed to grow to ± 80% confluency. To proceed with sample preparation, cells were fixed in 4% PFA, 2.5% glutaraldehyde (GA), 0.1 M Cacodylate buffer, followed by gradually increasing the ratio of culture media to fixative (1:3 1:1 3:1). When in fixative, cells were incubated for 3 h at room temperature with constant mild agitation. After fixation, cells were washed in cold 0.1 M cacodylate buffer and further incubated in 1% OsO_4_ at 4 °C, overnight with mild agitation. Cells were washed with cold ddH_2_O and left to incubate in 1% uranyl acetate (UrAc) for 1 h at 4 °C, in the dark. Following UrAc incubation, cells were dehydrated through incubation with an increasing gradient of EtOH (7%, 15%, 30%, 50%) and left to incubate overnight in 70% EtOH. The following day, cells were further incubated with 95% EtOH, 100% EtOH, and 100% anhydrous EtOH to finalize the dehydration process. After dehydration samples were infiltrated with Spurr’s resin with a gradual increase in concentration (1/3-2/3-3/3), with an incubation of at least 8 h per step. To finalize sample preparation, the cells were embedded in Spurr’s resin and allowed to polymerize overnight at 70 °C.

For EM imaging, resin blocks were sectioned to make ultrathin sections of gold interference colour with a Leica EM UC6 ultra-microtome. Following sectioning, samples were post-stained in a Leica EM AC20 for 40 min in UrAc at 20 °C, and for 10 min in lead citrate at 20 °C.

### Western blotting

Part of the muscle biopsies obtained and fibroblasts cultured from skin biopsies were manually homogenized in RIPA buffer containing both protease and phosphatase inhibitors while kept at 4 °C (on ice) to prepare protein extracts. The lysate was centrifuged and the supernatant collected. For SDS-PAGE each sample was prepared in non-reducing sample buffer (Invitrogen), with 80 mM DTT and heated at 95 °C for 5 min. Samples were loaded onto pre-cast 4–12% Bis–Tris polyacrylamide gels (Invitrogen) and proteins separated according to their molecular weight in MOPS running buffer at a voltage of 200 V and 500 mA of current. After electrophoresis, the gels were then transferred to a nitrocellulose membrane using a dry-transfer method using the iBlot2 gel transfer device (Invitrogen). The membrane was blocked with membrane blocking reagent (Cytiva) in TBST 2% (w/v). After blocking membranes were incubated with primary antibodies in blocking solution at the appropriate dilution (antibody table in supplementary table 5, online resource). Following primary antibody incubation, membranes were washed and incubated with the appropriate secondary antibody conjugated with horse-radish peroxidase (HRP) to allow the detection by chemiluminescence. For detection SuperSignal Dura ECL (Invitrogen) was used as a substrate. Membranes were imaged using a Bio-Rad Chemi-Doc imager device and the obtained images analyzed and quantified using the Image-Lab v6.1 software package (Bio-Rad, USA). After imaging, when required, the membranes were stripped using Restore western blot stripping buffer (Invitrogen), and then re-stained for other targets following the procedure described above.

### In vitro phase separation and aggregation assays

#### Cloning

pJ4M/TDP-43 was a gift from Nicolas Fawzi (Addgene plasmid # 104480; http://n2t.net/addgene: 104480; RRID:Addgene_104480). TDP-43^p.Trp385IlefsTer10^ was generated via Gibson assembly utilizing a PCR-linearized insert and a PCR-linearized vector of pJ4M/TDP-43, and verified with Sanger sequencing. TDP-43^p.M337V^ was generated via QuikChange site-directed mutagenesis according to manufacturer protocols (Agilent), and verified with Sanger sequencing.

#### Protein purification

TDP-43 was purified as described [21]. In brief, WT TDP-43, TDP-43^p.Trp385IlefsTer10^ and TDP-43^p.M337V^ expression plasmids were transformed into *Escherichia Coli* BL21-CodonPlus (DE3)-RIL competent cells (Agilent). Transformed *E. coli* were grown in small cultures in LB with kanamycin (50 μg/mL) and chloramphenicol (34 μg/mL) at 37 °C for 4 h. Cultures were transferred to 1L of LB media supplemented with both antibiotics and glucose (0.2% w/v), and grown at 37 °C until OD_600_~0.5. Protein expression was induced with 1 mM IPTG for 16 h at 16 °C. Cells were harvested by centrifugation, resuspended in resuspension/wash buffer (20 mM Tris–HCl pH 8.0, 1 M NaCl, 10 mM imidazole, 10% glycerol, 1 mM DTT, 5 μM Pepstatin A, 100 μM PMSF, and complete, EDTA-free, Roche Applied Science protease inhibitors), and lysed by lysozyme (1 mg/mL) and sonication. Cell lysates were centrifuged at 30,966 rcf at 4 °C for 20 min. Protein was purified over Ni–NTA resin (QIAGEN) and eluted from the resin with elution buffer (wash buffer except with 300 mM imidazole). The protein was further purified over amylose resin (NEB) and eluted with 20 mM Tris–HCl pH 8.0, 1 M NaCl, 10 mM imidazole, 10% glycerol, 1 mM DTT, 5 μM Pepstatin A, 100 μM PMSF, and 10 mM maltose. The protein was concentrated using Amicon Ultra-15 centrifugal filters, MWCO 50 kDa (Millipore), aliquoted, flash frozen in liquid nitrogen, and stored at −80 °C until further use.

#### In vitro phase separation assay

Full-length WT TDP-43, TDP-43^p.Trp385IlefsTer10^ and TDP-43^P.M337V^ MBP and His tags attached to the protein) was thawed on ice, then centrifuged at 21,300 rcf at 4 °C for 10 min. TDP-43 was then buffer exchanged into assay buffer (150 mM NaCl, 20 mM HEPES–NaOH pH 7.4, 1 mM DTT) according to manufacturer protocols (Bio-Rad Micro Bio-Spin Chromatography Columns). TDP-43 concentration was determined via NanoDrop (WT and TDP-43^p.M337V^: e_280_ = 114,250 Cm^−1^ M^−1^; TDP-43^p.Trp385IlefsTer10^. e_280_ = 108,750 cm^−1^ M^−1^). 20% Dextran and TDP-43 were then added to assay buffer to achieve final reaction conditions (5 μM TDP-43, 150 mM NaCl, 20 mM HEPES–NaOH pH 7.4, 0.5 mM DTT, 10% Dextran). Samples were incubated at room temperature (~25 °C) for 30 min. before subsequent assessment by microscopy, turbidity measurements, and C_sat_ determination. Brightfield microscopy was conducted on an EVOS M5000 utilizing a 100× objective. Turbidity measurements were conducted as one reading at absorbance 395 nm in a nonbinding 96-well plate (Greiner) in an Infinite M1000 Tecan plate reader. For data analysis, standardized turbidity values were calculated via subtraction of the reaction buffer turbidity value from the sample turbidity values. For C_sat_ determination, samples were centrifuged at 21,300 rcf at room temperature (~25 °C) for 10 min., followed by NanoDrop measurements of the supernatant to assess TDP-43 concentration as above.

### *Drosophila* studies

Fly lines were generated to express the different TDP-43 variants by using site-specific genomic integration using the attP2 landing site. *Drosophila* lines used for this study are listed in Supplementary Table 2 (online resource).

#### Rescue analysis

Offspring was collected daily and viability of all generated genotypes was registered. Results were compared to the expected ratio of offspring assuming a mendelian inheritance pattern.

#### Survival assays

Flies expressing the transgenes of interest, driven by the nSyb pan-neuronal driver, were generated by crossing line nSyb-Gal4 with TDP-43 WT, TDP-43 p.A382T, TDP-43 p.M337V and TDP-43 p.Trp385IlefsTer10. For the inducible driver Gal80 assays, tub-Gal80, Mef2-Gal80 and nSyb-Gal80 were used as the driver lines. To avoid crowding effects, for each condition flies were divided into groups of 10 individuals (males and females separate), except where there were insufficient numbers. Every 2–3 days, all groups were observed and deaths recorded. Flies unable to stand up or immobilized at the bottom of the vial were recorded as dead.

#### Climbing assays

The same flies generated for the nSyb-Gal4 survival assays (described above) were used for the climbing assays. For each condition, flies were divided in groups of 30 individuals (males and females separate), except where there were insufficient numbers. Motor performance was evaluated every week since the start of the assay. Climbing ability was determined by counting the number of flies that were successful in climbing past the 150 ml mark on a 250 ml glass cylinder within 15 s.

### Primary neuron transfections

Primary mixed cortical neurons were dissected from embryonic day 19–20 Long-Evans rat pups and cultured at 0.6 × 10^6^ cells/mL in 96 well cell culture plates (TPP), as previously described (Archbold et al. 2018; Malik et al. 2018; Weskamp and Safren et al. 2019). At in vitro day (DIV) 4, neurons were transfected with 0.2 μg DNA and 0.5 μL Lipofectamine 2000 (ThermoFisher) per well, per the manufacturer’s protocol, with the exception that cells were incubated with Lipofectamine/DNA complexes for only 20 min at 37 °C before rinsing.

### Longitudinal fluorescence microscopy and image analysis

Automated longitudinal fluorescence microscopy was performed as described previously [2, 3, 5, 20, 37, 39]. Images of cultured neurons were acquired by an inverted Nikon Ti microscope equipped with a ×20 objective lens, a Perfect-Focus system, a Lambda 421 multiLED light source with 5 mm liquid light guide (Sutter), and either an Andor iXon3 897 EMCCD camera or Andor Zyla4.2 ( +) sCMOS camera. All stage, shutter, and filter wheel movements were carried out by custom scripts written in the BeanShell environment within μManager [37, 62]. Image segmentation and survival analyses were accomplished through code scripts written in Python and the ImageJ macro language (as described in [37, 62]). Supplementary Fig. 5, online resource.

### Transcriptomics

Two patient muscle tissue samples (Fig. 1a: IV.4 and IV.5) along with three control samples were sent for RNA-sequencing (Macrogen Europe). The library preparation was done with an Illumina TruSeq Stranded Total RNA ribo-zero Gold kit, and the sequencing done on a NovaSeq6000 sequencer. Samples were run on a S4 flow cell at 50 M reads/sample, resulting in 150 bp paired-end reads. The resulting Fasta files were aligned using the STAR aligner [17] and the Samtools package [34] was used to generate indexes for the resulting BAM files. The QoRTs [24] java package was used to generate QC data and count files for the downstream analysis. Visualization of the alignments was done using the IGV software [47]. Differential gene expression analysis of the RNA-Seq data was done using the EdgeR v3.32.1 R package [48]. Significant differentially expressed genes were selected at the cut-off values log_2_fc ≥∣1∣ and adjusted *p* value ≤ 0.05. Differential gene expression was further analyzed to identify affected pathways and primary GO terms by using the Gene Set Enrichment Analysis (GSEA) software v4.2.2 [40, 54] and performing a Metascape analysis [63]. For analyzing alternative splicing (AS) patterns the rMATS turbo v4.1.2 computational tool was used [51]. rMATS detects the primary 5 different types of splicing events: alternative 3’ splice-sites (A3SS), alternative 5’ splice-sites (A5SS), skipped exons (SE), retained introns (RI) and mutually exclusive exons (MXE). Additionally, it computes the p-value and false discovery rate (FDR) of the ratio of isoforms between the two study conditions filtered by a user-defined difference threshold. For our analysis the threshold was left at the default setting of 0.0001 (0.01% splicing difference). AS events were selected as significant when the conditions FDR ≤ 0.01 and ∣Ψ∣≥ 0.1 were met.

### Ethics statement

All vertebrate animal work was approved by the Committee on the Use and Care of Animals (UCUCA) at the University of Michigan. All experiments were performed in accordance with UCUCA guidelines and designed to minimize animal use. Rats (*Rattus norvegicus*) were housed single in chambers equipped with environmental enrichment and cared for by veterinarians from the Unit for Laboratory Animal Medicine at the University of Michigan. All individuals were trained and approved in the care of long-term maintenance of rodents, in accordance with the NIH-supported Guide for the Care and Use of Laboratory Animals. All personnel handling the rats and administering euthanasia were properly trained in accordance with the University of Michigan Policy for Education and Training of Animal Care and Use Personnel. Euthanasia followed the recommendations of the Guidelines on Euthanasia of the American Veterinary Medical Association. Brains from individual pups in each litter were pooled to maximize cell counts prior to plating; as a result, primary cortical neurons used for all studies include an even mix of cells from both male and female pups.

## Results

### An extended Belgian family with an unsolved autosomal dominant rimmed vacuole myopathy

We report a family with 18 affected individuals in 5 successive generations, presenting with an unsolved autosomal dominant slowly progressive myopathy (Fig. 1a) (Supplementary Fig. 1, online resource). The family was initially identified more than 40 years ago. All family members came from the provinces of East and West Flanders in Belgium. The clinical data are summarized in Table 1 and a detailed description of the clinical characteristics of this family is provided in the Supplementary data, online resource. In summary, patients presented with weakness due to a myopathy with MRI imaging showing fatty infiltration and muscle atrophy (Fig. 1b) characterized by proximal and distal muscle weakness, predominantly of the lower limbs but over time also affecting the upper limbs. The mean age of onset was 34 ± 9 years and the disease led to wheelchair-dependence between 45 and 60 years of age. No FTD or ALS cases were reported in this family.

Muscle biopsies obtained from four patients (II.1, III.5, IV.4 and IV.5) confirmed the diagnosis of a rimmed vacuole myopathy. Histological evaluation of patient muscle showed the presence of centralized nuclei and a striking disparity in muscle fibre size (Fig. 1c, i-iii). Additionally, trichrome staining revealed the presence of rimmed vacuoles, along with increased infiltration of fatty tissue (Fig. 1c, iv-vi). TEM revealed the presence of an increased number of autophagosomal vesicles (Fig. 1c, vii), accumulation of fibrous material within the muscle fibres (Fig. 1c, viii) (Supplementary Fig. 2a (iii), online resource) and sporadic mitochondrial abnormalities (Supplementary Fig. 2a (ii), online resource). No changes were seen at the motor end plates, in line with a predominantly myopathic origin of the pathology (Supplementary Fig. 2a (i), online resource). In addition, we performed TEM in patient muscle-derived cultured myocytes and observed highly enlarged autophagosomes, presenting with an unusual “zebra” pattern, consistent with disturbed autophagy (Supplementary Fig. 2b, online resource). These results strongly suggest that the patients in this family are affected by a molecularly unexplained autosomal dominantly inherited condition showing the typical signs of a rimmed vacuole myopathy.

### Autosomal dominant rimmed vacuole myopathy is conclusively linked to a novel 11 bp deletion (c.1152_1162del) in TARDBP

**Fig. 2:**
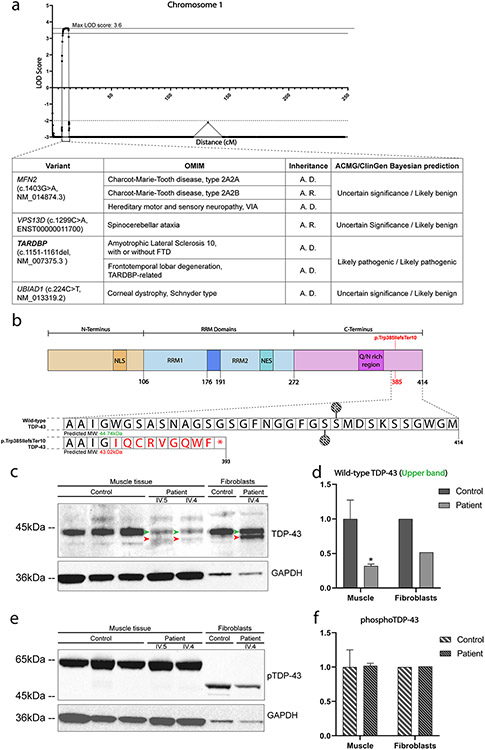
TDP-43^p.Trp385IlefsTer10^ protein is expressed in mutant muscle tissue and fibroblasts. **a** LOD-score plot of chromosome 1 showing conclusive linkage (Max. multipoint LOD Score = 3.61) to chromosome 1p36.22 at 8.1–13.8 cM along with the variants found in the region of interest. Further information on the classification of the variants can be found in Supplementary table 1. **b** Graphical representation of the structure of the TDp-43 protein and the changes to the protein sequence caused by TDP-43^p.Trp385IlefsTer10^. Phosphorylatable sites Ser409 and Ser410 (represented with a “P”) are lost in the mutant protein, meaning this phosphorylation marker is lost in TDP-43^p.Trp385IlefsTer10^. **c** TDP-43 levels in muscle tissue and fibroblasts collected from patients. Muscle of patients shows a clear reduction in TDP-43 levels, whereas TDP-43 levels in fibroblasts are evenly distributed between the wild-type (green arrowhead) and mutant (red arrowhead) forms of TDP-43. **d** Relative quantification of the levels of WT TDP-43 protein in patient tissues versus control (n_patient_ = 2, n_control_ = 3). Protein levels were normalised against GAPDH levels for all conditions. **e** Despite the altered levels of TDP-43 in muscle, the levels of phosphorylated TDP-43 remain unaffected in both muscle and fibroblasts. **f** Relative quantification of the levels of phosphorylated TDP-43 protein in patient tissues versus control (n_patient_ = 2, n_control_ = 3). Protein levels were normalised against GAPDH levels for all conditions. Statistical analysis performed using the standard twoway ANOVA test using multiple comparisons; *p < = 0.05

To identify the underlying genetic defect in this family, we combined linkage analysis and WES. Genome-wide multipoint linkage analysis was performed using SNP-array data from 10 family members (9 affected, 1 unaffected). We obtained conclusive linkage with a maximum multipoint logarithm of the odds (LOD)-score of 3.61 at marker rs3010876, located at chromosome 1p36.22 (Fig. 2a). Haplotype analysis identified a 4.7 Mb disease haplotype between markers rs10867739 and rs10927466 that was shared between all affected family members (Supplementary Fig. 2c, online resource). No other genomic regions with a LOD-score > 3.2 were identified (Supplementary Fig. 3, online resource). WES analysis in patient IV.4 identified a heterozygous 11 bp deletion (c.1152_1162del) in exon 6 of the *TARDBP* gene, located within the linked 4.7 Mb interval at 1p36.22 (Fig. 2a) and predicted to induce a frameshift and premature termination codon (TDP-43^p.Trp385IlefsTer10^, Fig. 2b). The presence of this variant was confirmed in all affected individuals by Sanger sequencing. The variant was absent in the gnomAD database (v2.1.1) and has not been reported previously. Three other variants in Mendelian disease genes *MFN2*, *VPS13D* and *UBIAD1* were found in the linked region (Fig. 2a, table) but were not considered pathogenic based on established variant classification tools (Supplementary table 1, online resource) [46]. These results strongly suggest *TARDBP* c.1152_1162del as the pathogenic variant causing myopathy with rimmed vacuoles in this family.

### TDP-43^p.Trp385IlefsTer10^ is expressed in patient-derived muscle and accompanied by reduced WT TDP-43 levels

*TARDBP* c.1152_1162del is predicted to produce a TDP-43 protein with a truncated and altered C-terminal PrLD (TDP-43^p.Trp385IlefsTer10^). To evaluate whether *TARDBP* c.1152_1162del containing mRNA transcripts indeed escape elimination by nonsense-mediated decay (NMD), we analyzed the RNA sequencing (RNA-seq) data of patient-derived muscle tissue. This showed that ~ 55% of the reads bridging position c.1152_1162 contained the expected 11 bp deletion (Supplementary Fig. 4, online resource). Western blot analysis further confirmed the presence of a lower band in both patient-derived muscle tissue and fibroblasts (Fig. 2c, red arrowheads) indicating that the mutant transcripts are not removed by NMD and are translated into the predicted truncated TDP-43^p.Trp385IlefsTer10^. Full-length TDP-43 levels produced by the wild-type (WT) allele were clearly reduced in patient-derived muscle, beyond the expected 50%, while in fibroblasts this was closer to the expected 50% (Fig. 2c, d). Interestingly, levels of TDP-43 phosphorylated at residues Ser_[409/410]_ (which are absent in the mutant protein), were normal in patient-derived muscle and fibroblasts (Fig. 2e, f). These results indicate that, in muscle, *TARDBP* c.1152_1162del leads to the expression of a truncated TDP-43^p.Trp385IlefsTer10^ protein along with clearly reduced full-length WT TDP-43 but normal Ser_[409/410]_ phosphorylated WT TDP-43 levels. In patient fibroblasts however, a different pattern was observed, with WT TDP-43 levels ~ 50% and Ser _[409/410]_ phosphorylated WT TDP-43 migrating as a lower molecular weight band (~ 50 kDa) compared to muscle (~ 65 kDa) suggesting tissue-specific differences in protein modification and expression.

### TDP-43 accumulations found in patient muscle mainly consist of mutant protein

**Fig. 3:**
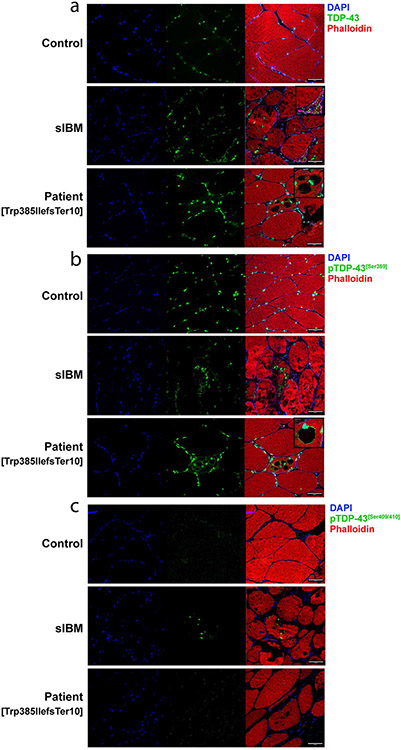
Phosphorylation state of TDP-43^p.Trp385IlefsTer10^ accumulation suggests a predominantly mutant protein composition. Immunofluorescent staining of control, sIBM and patient muscle tissue. **a** TDP-43 shows primarily nuclear staining, with presence of TDP-43 accumulation in sIBM and Patient tissue (insets). **b** Staining for pTDP-43[Ser369] reveals that TDP-43 accumulations are positive for phosphorylation of Ser396 in both sIBM and TDP-43^p.Trp385IlefsTer10^ patient tissues. **c** This is not the case for pTDP-43[Ser409/410], where only sIBM tissues have TDP-43 accumulation positive for this phosphorylation marker. Scale bar = 50 μm

Since rimmed vacuole myopathies are characterized by TDP-43-positive sarcoplasmic accumulations and nuclear depletion of TDP-43, we performed immunofluorescent staining on cryosections of muscle tissues obtained from patients. These types of inclusions are also known to be a prominent pathological feature in sIBM, so sIBM patient tissue was included as a positive control. We observed the presence of TDP-43-positive sarcoplasmic accumulations in both sIBM and TDP-43^p.Trp385IlefsTer10^ patients (Fig. 3a, insets), which were absent in control tissue. These accumulations were also positive for p62 (data not shown). Additionally, TDP-43 accumulations in sIBM and TDP-43^p.Trp385IlefsTer10^ tissues were positive for phosphorylation of the Ser369 residue, which also prominently labelled the rims surrounding the vacuoles of TDP-43^p.Trp385IlefsTer10^ tissue (Fig. 3b, inset). TDP-43 phosphorylated at residues Ser409 and Ser410 was observed in accumulations found in sIBM patients, but not found in TDP-43^p.Trp385IlefsTer10^ patient tissue (Fig. 3c). Finally, TDP-43 accumulations in TDP-43^p.Trp385IlefsTer10^ muscle tissue are often DAPI-positive indicating that they contain nucleic acids (Fig. 3a,b). The results indicate that the composition of the TDP-43 accumulations found in TDP-43^p.Trp385IlefsTer10^ patients differs from that of the ones found in sIBM patients. The absence of pTDP-43[Ser409/410]-positive accumulations in patient tissues suggests that the protein composition of the pTDP-43[Ser369]-positive accumulations excludes the wild-type form of TDP-43 and mainly consists of the mutant variant lacking the Ser409 and Ser410 residues.

### TDp-43^p.Trp385iiefsTer10^ is aggregation-prone compared to WT TDP-43

**Fig. 4:**
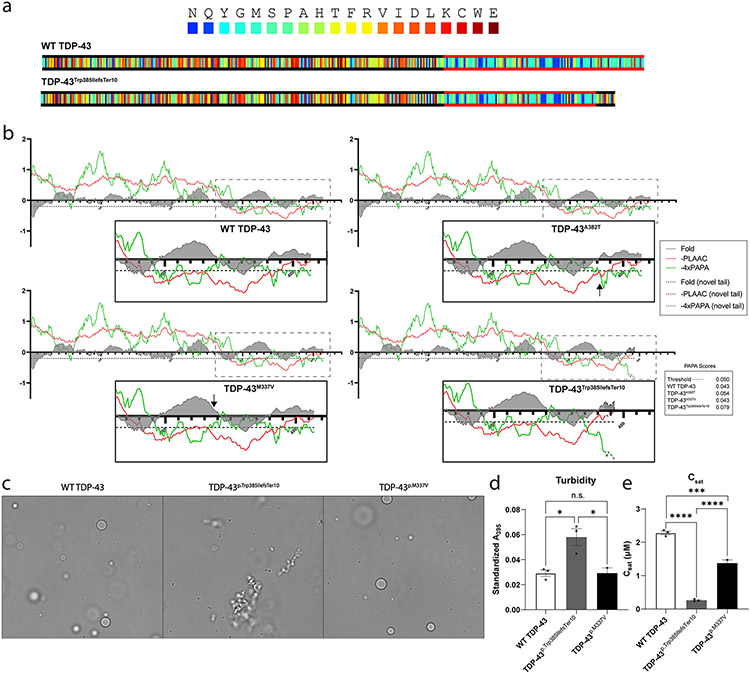
TDP-43^43p.Trp385IlefsTer10^ is aggregation-prone compared to WT TDP-43 and TDP-43^p.M337V^. **a-b** PLAAC prediction for prion-like domains shows that the PrLD of TDP-43^p.Trp385IlefsTer10^, marked with red lines in the color-coded sequence visualizations (**a**), and adjusted PAPA and negative PLAAC scores (**b**), spans a smaller region when compared to wild-type TDP-43 and the two ALS mutants. The black lines in the color-coded sequence (**a**) indicate that the aa. frequencies correspond to the background frequencies and hence no PrLD is identified. Each aa. is color-coded by its enrichment log-likelihood ratio in PrLDs (blue = high, red = low). The default settings (Lcore = 60 and S. cerevisiae background frequencies) were used to run the PLAAC application. Despite the smaller length in PrLD, PAPA predictions (green lines in **b**), indicate a higher propensity for aggregative potential in the mutant variant. The dashed line in **b** marks the threshold of the PAPA-score for prion-like behaviour. Minimal differences in Fold Index (FI), a prediction to identify intrinsically unfolded protein regions (= negative score), are observed between wild type TDP43, ALS TDP-43 mutants and PrLD of TDP-43^p.Trp385IlefsTer10^ **c-e** WT TDP-43-MBP-His and TDP-43^p.Trp385IlefsTer10^-MBP-His and TDP-43^p.M337V^-MBP-His (5 μM) were incubated for 30 min at room temperature. **c** Representative brightfield microscopy images indicate that WT TDP-43 and TDP-43^p.M337V^ form spherical droplets, whereas TDP-43^p.Trp385IlefsTer10^ forms irregularly shaped aggregates. The scale bar represents 10 μm. ***d*** Standardized turbidity, measured in a plate reader at 395 nm, is significantly higher for TDP-43^p.Trp385IlefsTer10^ versus WT TDP-43 and TDP-43^p.M337V^. Bars represent means ± SEM (*n* = 3), and each individual data point is shown. An ordinary one-way ANOVA with Tukey’s multiple comparisons test was performed, *p < 0.05. **e** The saturation concentration (C_sat_) of each sample was measured after centrifugation. C_sat_ is significantly lower for TDP-43^p.Trp385IlefsTer10^ versus WT TDP-43 and TDP-43^p.M337V^. Bars represent means ± SEM (*n* = 3), and each individual data point is shown. Statistical analysis performed using an ordinary one-way ANOVA with Tukey’s multiple comparisons test, ***p < 0.001, ****p < 0.0001

Since the C-terminal PrLD renders TDP-43 intrinsically condensation- and aggregation-prone, we wanted to compare the phase separation properties of WT TDP-43, TDP-43 ALS mutants and TDP-43^p.Trp385IlefsTer10^. We first used the in silico prediction tool PLAAC (Prion-Like Amino Acid Composition) [33], to identify the localisation and length of the PrLD, which includes a re-implementation of PAPA (Prion Aggregation Prediction Algorithm) [56] that allows the comparison of the predicted aggregation propensities. While the PrLD of TDP-43^p.Trp385IlefsTer10^ was shown to be slightly shorter than that of the WT TDP-43, the maximum aggregation propensity score was ~ two-fold higher for TDP-43^p.Trp385IlefsTer10^ (PAPA score: 0.079) than for WT TDP-43 (PAPA score: 0.043) (Fig. 4b). TDP-43^p.M337V^ and TDP-43^p.A382T^ did not show such pronounced alterations to the maximum PAPA score, with only TDP-43^p.A382T^ increasing by ~ 25% (PAPA score: 0.054).

Next, we assessed the condensation properties of WT TDP-43, TDP-43^p.M337V^ and TDP-43^p.Trp385IlefsTer10^ utilizing in vitro phase separation assays with recombinant protein [21]. Brightfield microscopy images revealed morphological differences in WT TDP-43 and TDP-43^p.Trp385IlefsTer10^ condensates (Fig. 4c). WT TDP-43 forms spherical droplets, indicating that these condensates are liquid-like (Fig. 4c, left). Similarly, TDP-43^p.M337V^ also condensates into spherical droplets (Fig. 4c, right). By contrast, TDP-43^p.Trp385IlefsTer10^ forms irregular aggregated structures, including fibrillar forms, indicating a transition to solid-like condensates (Fig. 4c, center). TDP-43^p.Trp385IlefsTer10^ assemblies displayed increased turbidity compared to WT TDP-43 and TDP-43^P.M337V^ condensates, indicating a greater propensity for TDP-43^p.Trp385IlefsTer10^ to form higher order structures (Fig. 4d). We next measured the saturation concentration (C_sat_) for WT TDP-43, TDP-43^p.M337V^ and TDP-43^p.Trp385IlefsTer10^. C_sat_ is the concentration of protein in the soluble phase of a phase-separated system and provides a measure of the propensity of a protein to undergo phase separation. TDP-43^p.Trp385IlefsTer10^ has a significantly reduced C_sat_ value compared to WT TDP-43 and TDP-43^p.M337V^, indicating that TDP-43^p.Trp385IlefsTer10^ is more prone to phase separation than either the wild-type or the ALS-associated mutant protein (Fig. 4e). In summary, our in vitro findings are in line with the in silico predictions and suggest that TDP-43_p.Trp385IlefsTer10_ more readily undergoes phase separation to more solid-like condensates compared to WT TDP-43 and TDP-43^p.M337V^.

### TDP-43^p.Trp385IlefsTer10^ rescues the *TBPH* null pupal lethality but displays strongly reduced gain-of-function toxicity in *Drosophila*

**Fig. 5:**
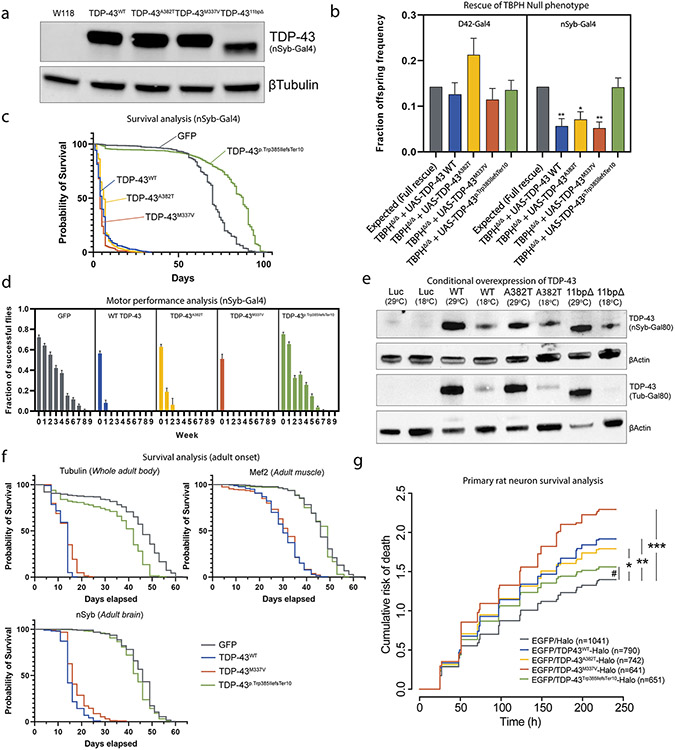
TDP-43^p.Trp385IlefsTer10^ behaves as a partial loss-of-function allele with reduced toxicity in *Drosophila*. **a** Expression levels of human TDP-43 in control and transgenic fly lines using the pan-neuronal nSyb-Gal4 driver. **b** Rescue assay results showing the rate of offspring frequency in *TBPH* null flies rescued with the TDP-43 constructs of interest driven by the nSyb-Gal4 pan-neuronal driver (right) and the D42-Gal4 motor neuron-specific driver (left). **c** Combined survival assay performed on overexpression models of the TDP-43 variants of interest driven by the nSyb-Gal4 neuronal driver. **d** Combined climbing assay performed on transgenic models of the TDP-43 variants of interest driven by the nSyb-Gal4 pan-neuronal driver. Fraction of flies capable of completing the assay was plotted weekly throughout the course of the assay. **e** Expression levels of human TDP-43 in control and transgenic fly lines using the nSyb-Gal80 inducible driver and the tubulin-Gal80 inducible driver. For the inducible drivers, the levels of protein expression at 29 °C vs. 18 °C confirm that the system is reliably inducing higher levels of expression when flies are kept at a higher environmental temperature (29 °C). **f** Combined survival results for both male and female transgenic and control flies with variant adult onset expression driven in a tissue-specific manner. From left to right: whole body expression with Tubulin-Gal80, muscular expression with Mef2-Gal80 and pan-neuronal expression with nSyb-Gal80. Expression of hTDP-43 variants was induced by moving the flies into a 29 °C environment. **g** Rodent mixed cortical primary neurons were transfected with the indicated constructs and tracked by automated fluorescence microscopy. Neuronal survival and time of death was assessed using objective criteria (described in Supplementary Fig. 4, online resource), and cumulative risk of death plotted for neurons in each condition. *N* = number of neurons, combined from 3 biological replicates. *hazard ratio (HR) = 1.29, *p* = 6.7×10^−7^; **HR = 1.31, *p* = 8.0×10^−8^; ***HR = 1.56, *p* < 2 × 10^−16^; ^#^HR = 1.17, *p* = 0.003. HR and *p* value determined by cox proportional hazards analysis, stratifying among biological replicates. Statistical analysis performed using the one-way ANOVA test (Kruskal-Wallis test); *p ≤ 0.05, **p ≤ 0.01 (unless otherwise stated)

To assess whether TDP-43^p.Trp385IlefsTer10^ displays loss- and/or gain-of-function properties in vivo, we generated fly lines with site-specifically integrated transgenic constructs containing WT or mutant TDP-43. In addition to WT TDP-43 and our novel TDP-43^p.Trp385IlefsTer10^, we chose two typical ALS-causing mutations located in the PrLD (TDP-43^p.A382T^ and TDP-43^p.M337V^). Using the pan-neuronal nSyb-GAL4 driver, the transgenic lines exhibited similar levels of expression (Fig. 5a). First, we performed rescue experiments to investigate potential loss-of-function properties of TDP-43^p.Trp385IlefsTer10^ therefore expressed the TDP-43 transgenes in flies lacking the endogenous *TARDBP* ortholog called *TBPH*. We showed that *TBPH* null flies display strongly reduced eclosion rates due to late-pupal lethality caused by loss of functional TBPH protein in neurons [12, 16]. Motor neuron-specific substitution of *TBPH*, utilizing the D42-GAL4 driver, with all human variants completely rescued the null lethal phenotype (Fig. 5b, left). Pan-neuronal substitution with TDP-43^p.Trp385IlefsTer10^ resulted in a complete rescue, whereas the rescue was only partial with WT TDP-43, TDP-43^p.A382T^ and TDP-43^p.M337V^ (Fig. 5b, right). We suspected these results were due to increased toxicity upon overexpression of WT TDP-43, TDP-43^p.A382T^ and TDP-43^p.M337V^ at supraphysiological levels. Indeed, neuronal overexpression of WT and ALS mutant TDP-43 in neurons of WT *TBPH* flies severely reduced motor performance and shortened the average lifespan of the flies, recapitulating previously published results [22]. However, and surprisingly, neuronal overexpression of TDP-43^p.Trp385nefsTer10^ did not display these neuronal toxic gain-of-function phenotypes. Flies overexpressing TDP-43^p.Trp385IlefsTer10^ in their nervous system had no significantly altered average survival nor motor performance compared to control flies (Fig. 5c) (Supplementary table 3, online resource). To investigate whether the absence of gain-of-function properties was tissue-specific, we expressed all human variants in the whole adult body, the adult brain and muscle. To circumvent developmental toxicity, we used the GAL80 system, allowing us to switch on the expression in adult flies. Protein levels were checked by western blot. All variants were silenced during development and switched on in adult flies to a similar extent (Fig. 5e). In none of the tested conditions gain-of-function toxicity of the TDP-43^p.Trp385IlefsTer10^ was observed, in contrast to WT TDP-43 and TDP-43^p.M337V^ (Fig. 5f) (Supplementary table 4, online resource). Altogether these *Drosophila* results show that, while retaining sufficient activity in neuronal context to rescue the lethal neurodevelopmental null phenotype, TDP-43^p.Trp385nefsTer10^ loses its toxic gain-of-function properties upon overexpression in WT *TBPH* backgrounds and thus behaves very differently from full-length WT or ALS-mutant TDP-43.

### The reduced gain-of-function toxicity of TDP-43^Trp385IlefsTer10^ is recapitulated in rat-derived primary neuronal culture

Following the observation that TDP-43^p.Trp385IlefsTer10^ displays lower neuronal toxicity compared to wild-type and ALS mutant variants when expressed in fruit fly models, we modelled the same variants using a primary rat neuron disease model. Primary cortical rat neurons were transfected with halo-tagged constructs expressing the TDP-43 variants of interest and fluorescently labelled neurons were tracked over time by using automated fluorescence microscopy (Supplementary Fig. 5, online resource). Neuronal death throughout the timed assay was assessed and registered to evaluate the cumulative risk of death of each TDP-43 variant.

TDP-43^p.Trp385IlefsTer10^ scored the lowest of all TDP-43 variants in study with a hazard ratio (HR) below the threshold of significance when compared to the mock transfection (Fig. 5g, EGFP/Halo). All other variants scored a significantly higher HR than the mock, with TDP-43^p.M337V^ having the highest HR value (HR = 1.56). This indicates that all TDP-43 variants display a significantly higher toxicity in neurons, leading to increased cell death when compared to TDP-43^p.Trp385IlefsTer10^. This pattern of increased toxicity, even for WT TDP-43 expression in neurons, recapitulated the results obtained in our *Drosophila* experiments, reinforcing that TDP-43^p.Trp385IlefsTer10^ has a decreased neuronal toxicity effect and is distinct from ALS-causing mutations.

### TDP-43^p.Trp385IlefsTer10^ causes altered splicing patterns of pre-mRNAs encoding sarcomeric proteins in patient-derived muscle

**Fig. 6:**
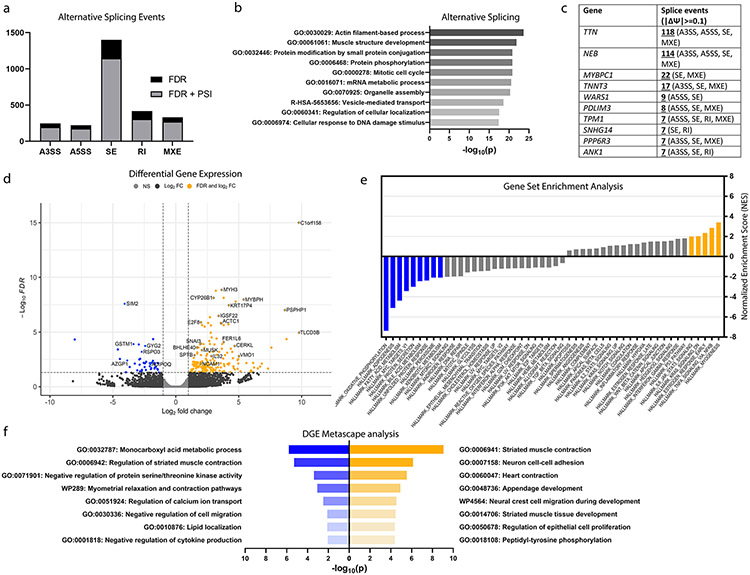
Abnormal splicing of sarcomeric genes, increased expression of muscle regeneration genes and decreased expression of mitochondrial and lipid metabolism genes in TDP-43^p.Trp385IlefsTer10^ muscle-derived transcriptomes. **a** Distribution of the number of alternative splicing events identified across each AS event type filtered by FDR ≤ 0.01 (FDR) and FDR ≤ 0.01 +∣ΔΨ∣≥ 0.1 (FDR + PSI). **b** Metascape analysis and top GO terms associated with the aggregated genes suffering AS events. **c** Top genes with multiple alternative splicing events identified and the respective types. Inclusion level difference filtered at ∣ΔΨ∣≥ 0.1. **d** Volcano plot of log_2_[fold change] values against the respective FDR values. Cutoff values for FDR ≤ 0.05 and ∣log_2_FC∣> 1. **e** Pathways identified by pre-ranked GSEA of the differentially expressed genes dataset and ranked by normalized enrichment score (NES). Pathways identified were considered significant at an FDR ≤ 0.01 (highlighted; blue—downregulated, gold—upregulated). **f** Metascape analysis of the top GO terms associated with the downregulated (blue) and upregulated (gold) genes identified in the DGE dataset

Given the well-established role of TDP-43 in pre-mRNA splicing, we analysed our RNA-seq datasets from muscle biopsies of two TDP-43^p.Trp385IlefsTer10^ patients and three controls to identify alternative splicing (AS) events as a readout of altered nuclear TDP-43 function. We used the rMATS turbo v4.1.2 computational tool which detects 5 different types of splicing events: alternative 3’ splice-sites (A3SS), alternative 5’ splice-sites (A5SS), skipped exons (SE), retained introns (RI) and mutually exclusive exons (MXE). rMATS identified a total of 2619 AS events at FDR ≤ 0.01, without Ψ filtering. We decided to select values at a threshold of inclusion of at least 10% (∣Ψ∣≥ 0.1). At ∣Ψ∣≥ 0.1 there were a total of 2096 AS events mapping to 1491 unique genes (Fig. 6a). Over half of the total AS events (55.03%) represented SE events. A Metascape analysis [63] of the list of genes with AS events highlighted the GO terms “Actin filament-based process” (GO:0,030,029) and “Muscle structure development” (GO:00,061,061) as the two most highly represented terms (Fig. 6b). Accordingly, out of the top 10 genes with the highest incidence of AS events, 6 indeed corresponded to important sarcomere filament proteins, including Titin (*TTN*), Nebulin (*NEB*), Myosin Binding Protein C1 (*MYBPC1*), Troponin T (*TNNT3*), Actinin-Associated LIM Protein (*PDLIM3*) and Tropomyosin 1 (*TPM1*), with the first two showing more than 100 individual AS events (Fig. 6c). Interestingly, all of these genes are associated with genetic (cardio)myopathies in humans (*TTN*, *NEB*, *MYBPC1*, *TNNT3* and *TPM1*) [18] or mice (*PDLIM3*) [43]. These data suggest that abnormal nuclear pre-mRNA splicing of functionally important sarcomeric genes could contribute to the pathogenic mechanism of the myopathy in our TDP-43^p.Trp385IlefsTer10^ family.

### TDP-43^p.Trp385IlefsTer10^ mutant muscle shows increased expression of muscle regeneration genes and decreased expression of mitochondrial and lipid metabolism genes

Next, we used EdgeR (v3.32.1) to identify differentially expressed genes (DEGs) as a consequence of TDP-43^p.Trp385IlefsTer10^ in our RNA-seq dataset. Out of the 28,983 total genes detected, 217 genes were identified which were significantly up- (*n* = 173) or down-regulated (*n* = 44) in patient muscle (∣log_2_fc∣≥ 1, FDR ≤ 0.05) (Fig. 6d). To find a potential link between splicing alterations identified by the rMATS tool and gene expression levels, we filtered our dataset to extract significantly up- and down-regulated genes (FDR ≤ 0.05) that intersected with genes showing significant AS events (FDR ≤ 0.01, ∣Ψ∣≥ 0.1). This resulted in a total of 15 genes that displayed both AS and differential expression (< 1% overlap) (Data not shown). Interestingly, these did not include *TTN*, *NEB* or any of the top 10 genes with the highest number of AS events, suggesting that their aberrant splicing does not directly result in altered transcript levels.

To identify TDP-43^p.Trp385IlefsTer10^-mediated dysregulated functional pathways in a more general manner, we performed Gene Set Enrichment Analysis (GSEA) [45, 54] of our RNA-seq dataset using the collection of 40 Molecular Signatures Database hallmark gene sets [35]. GSEA is a threshold-free method that is recommended when ranks are available for all or most of the genes in the genome and searches for pathways whose genes are enriched at the top or bottom of the ranked list. We therefore generated a pre-ranked list of 21,797 genes based on the −log_10_(p-value) multiplied by the sign of fold-change. GSEA showed that genes of the “myogenesis” hallmark gene set were strongly enriched among the upregulated genes at the top of our list (Fig. 6e, yellow). The bottom of the ranked list was strongly associated with “oxidative phosphorylation”, “adipogenesis” and “fatty acid metabolism” hallmark gene sets (Fig. 6e, blue). Of note, some of the most highly ranked upregulated DEGs in the core enrichment “myogenesis” gene set encoded sarcomere-associated myosin isoforms. In particular, we found myosin heavy chains 3 (*MYH3*) and 8 (*MYH8*). As embryonic myosin isoforms, *MYH3* and *MYH8* are expressed almost exclusively during development, being only re-expressed during muscle regeneration [49]. Their upregulation in patient muscle indicates an activation of regenerative mechanisms, which is typically seen in myopathies. As a confirmation of the GSEA pathways, we observed that a Metascape analysis applied to the statistically significant DEGs of our dataset highlighted the GO term “Striated muscle contraction” (GO:0,006,941) as being highly represented among the upregulated set of genes set (Fig. 6f, yellow) and “Monocarboxylic acid metabolic process” (GO:0,032,787) among the downregulated one (Fig. 6f, blue). Together, the transcriptomic AS and DEG analysis show that TDP-43^p.Trp385IlefsTer10^ mutant muscle bears the signature of a myopathy with dysregulated mitochondrial and lipid metabolism, possibly as a result of the aberrantly spliced sarcomere genes.

## Discussion

While neuropathological and genetic studies have established the crucial involvement of TDP-43 proteinopathy in the pathogenesis of ALS/FTD and related neurodegenerative disorders [57], increasing evidence also suggests a role for TDP-43 in muscular disorders, including sIBM and other rimmed vacuole myopathies [58]. However, genetic evidence supporting a primary role for TDP-43 proteinopathy in rimmed vacuole myopathies is currently missing. The present study reports the discovery of a frameshift variant, TDP-43^p.Trp385IlefsTer10^, producing a truncated and altered C-terminal PrLD of TDP-43 in a family presenting with an autosomal dominantly inherited rimmed vacuole myopathy. Even though this variant has only been identified in one large family, the pathogenic nature of TDP-43^p.Trp385IlefsTer10^ is supported by multiple lines of genetic evidence: it co-segregated in all affected members of a large myopathy family, it mapped to the sole conclusively linked genomic region in this family at chromosome 1p36.22 (maximum multipoint LOD-score 3.61), it was absent from the gnomAD population database and it was observed in the absence of other known genetic causes of rimmed vacuole myopathy. In addition, the TDP-43^p.Trp385IlefsTer10^ protein was expressed in affected patient-derived tissue and associated with the presence of TDP-43-positive sarcoplasmic accumulations in muscle, a common feature in rimmed vacuole myopathies [31]. Of interest, we found that these TDP-43 accumulations in TDP-43^p.Trp385IlefsTer10^ patients differ from those in sIBM patients. Where both are positive for phosphorylation of the Ser369 residues, only sIBM accumulations are positive for phosphorylation of Ser409 and Ser410, a commonly used disease-specific marker for TDP-43 proteinopathies. This discrepancy in composition of phosphorylated forms of TDP-43 between sIBM and TDP-43^p.Trp385IlefsTer10^ patients could naturally be attributed to the lack of the Ser409/410 residues in the mutant TDP-43^p.Trp385IlefsTer10^ protein. Interestingly, TDP-43^p.Trp385IlefsTer10^ patient tissues still show high levels of pTDP-43[Ser409/410], even being at equal levels compared to control muscle tissue. These data suggest that accumulations in TDP-43^p.Trp385IlefsTer10^ muscle tissue are primarily composed of mutant protein, while pTDP-43[Ser409/410]—originating from the wild-type allele—remains at baseline levels in a soluble state.

Strikingly, with its neomorphic tail of 9 aa. following the slightly shortened PrLD, TDP-43^p.Trp385IlefsTer10^ clearly differs from the ALS-causing missense variants which are present in an otherwise normal PrLD. Indeed, a major and unexpected finding in this study was the mainly chronic and slowly progressive myopathic clinical manifestation associated to TDP-43^p.Trp385IlefsTer10^. Although milder neurogenic signs were also present in some patients, ALS and/or FTD phenotypes were never observed. The specific nature of our truncating variant therefore likely holds the key as to how TDP-43^p.Trp385IlefsTer10^ leads to a muscular rather than a neurodegenerative condition. Interestingly, recent reports have linked a similar truncating TDP-43 variant, TDP-43^p.Y374Ter^, to a typical ALS phenotype [13, 14, 30]. Although the pathogenic nature of TDP-43^p.Y374Ter^ is not yet firmly established, it lacks the neomorphic 9 aa. tail present in TDP-43^p.Trp385IlefsTer10^. To this point, it is of interest that TDP-43^p.Trp385IlefsTer10^ behaved completely differently compared to WT TDP-43 or TDP-43^p.M337V^ in in vitro phase separation assays. In contrast to WT TDP-43 and TDP-43^p.M337V^, which are both able to form well-defined spherical droplets, TDP-43^p.Trp385IlefsTer10^ appeared to quickly aggregate with the formation of dense, misshapen accumulations of protein. This observation is in line with the PAPA analysis predicting a ~ two-fold higher maximum aggregation propensity score at the neomorphic C-terminus of TDP-43^p.Trp385IlefsTer10^ (PAPA score: 0.079) compared to WT TDP-43 or TDP-43^p.M337V^ (PAPA score: 0.043). Of note, the reported heterozygous ALS-associated TDP-43^p.Y374Ter^ truncation variant [13, 14, 30] scores lower than WT TDP-43 in the same prion prediction in silico tools (PAPA-score of 0.036) (data not shown), further suggesting an important role of the novel 9 aa. tail of TDP-43^p.Trp385IlefsTer10^ in inducing a myopathic rather than a neurodegenerative presentation.

Since the phase separation studies indicated increased aggregation propensity of TDP-43^p.Trp385IlefsTer10^, we were surprised to see that TDP-43^p.Trp385IlefsTer10^ exhibited strongly reduced toxic gain-of-function effects compared to WT TDP-43 and ALS-mutant TDP-43 in *Drosophila* survival and motor performance assays using different drivers. On the other hand, TDP-43^p.Trp385IlefsTer10^ was capable of rescuing the *Drosophila TBPH* null lethal phenotype when driven in neuronal tissues, indicating that the mutant protein retains considerable normal activity. TDP-43^p.Trp385IlefsTer10^ therefore behaves as a partial loss-of-function allele in *Drosophila* with strongly reduced toxic gain-of-function properties. We were able to confirm that this reduced toxic gain-of-function is still seen in primary rat cortical neurons, which displayed a pronounced reduction in lethality when comparing TDP-43^p.Trp385IlefsTer10^ and WT or ALS TDP-43 overexpression. These findings are in agreement with the very high pLI-score of 0.99 of *TARDBP* (https://gnomad.broadinstitute.org/) indicating that it is intolerant of heterozygous loss-of-function variants or haploinsufficiency [64]. In this scenario, the residual function of TDP-43^p.Trp385IlefsTer10^ would be sufficient to support viability but not muscle health in humans. How and whether the aggregation and partial loss-of-function properties of TDP-43^p.Trp385IlefsTer10^ are linked in flies is currently unclear. One explanation is that the WT TDP-43-mediated overexpression toxicity is not aggregation-dependent, and that TDP-43^p.Trp385IlefsTer10^ possibly loses its toxicity because aggregation renders it inert, thereby reducing its activity. This interpretation would be in line with the idea that the formation of insoluble TDP-43-aggregates is not directly harmful and that cellular toxicity is instead mediated by dynamic liquid-like condensates [8]. It could also explain why the strongly aggregation-prone TDP-43^p.Trp385IlefsTer10^ is less toxic to neurons and does not cause a neurodegenerative ALS/FTD phenotype.

Despite our functional studies suggesting that TDP-43^p.Trp385IlefsTer1^ acts as an aggregation-prone partial loss-of-function variant with decreased toxicity, this does not explain how this would preferentially affect muscle tissue in humans. It is of interest that we observed differences in TDP-43 expression and phosphorylation in muscle versus fibroblasts from TDP-43^p.Trp385IlefsTer10^ patients and controls. In particular, our data suggest that, despite the presence of TDP-43^p.Trp385IlefsTer10^, which lacks the normal TDP-43 residues 385–414, normal levels of Ser_[409/410]_ phosphorylated TDP-43 are maintained in muscle tissue, apparently at the expense of unmodified full-length WT TDP-43 from the normal allele, which could further contribute to a depletion and loss-of-normal function of WT TDP-43. Remarkably, this phenomenon was not observed in fibroblasts, reinforcing the idea that TDP-43 undergoes muscle-specific processing and modification including phosphorylation, thereby providing a possible explanation of the myopathy phenotype associated with TDP-43^p.Trp385IlefsTer10^. Further evidence for a tissue-specific function of TDP-43 was recently obtained in mouse muscle and neuronal cells showing cell-type-specific differences in TDP-43-mediated pre-mRNA processing [55].

In line with the proposed partial loss-of-function nature of TDP-43^p.Trp385IlefsTer10^, our transcriptomic analysis of TDP-43^p.Trp385IlefsTer10^ muscle tissue provided evidence for a nuclear loss-of-function. Through analysis of alternative pre-mRNA processing patterns, a nuclear process TDP-43 is tightly involved in, we identified a number of transcripts affected by AS. Interestingly, the great majority of these AS targets corresponded to muscle-specific structural/contraction-associated proteins. Indeed, 5 out of the top 10 transcripts with the most AS events corresponded to sarcomeric genes, including *TTN* and *NEB*, indicating that abnormal nuclear pre-mRNA splicing of functionally important sarcomeric genes could contribute to the pathogenic mechanism of the myopathy in our family.

In mice, TDP-43 has been shown to associate with sarcomeric mRNAs, such as *TTN* and *NEB*, within sarcoplasmic myo-granules during muscle development/regeneration [59], and is hypothesized as having an important role for the stabilization of these transcripts. Curiously, when comparing differentially expressed genes with genes affected by AS, we observed a very small overlap (< 1%), suggesting that aberrant splicing in the nucleus is not having a direct effect in the cellular expression and stability of these transcripts, apparently having a functional impact instead. Pathway analysis of the upregulated genes highlighted myogenesis and striated muscle contraction-associated genes as strongly altered in patient muscle. Vogler et. al [59] showed that heterozygous TDP-43 KO mice are unable to successfully regenerate muscle fibres after injury. Though still in need of molecular validation, if we assume a similar effect is occurring with the TDP-43^p.Trp385IlefsTer10^, the attempted and failed regeneration by the muscle fibres would result in an upregulation of myogenic pathways and certain sarcomeric mRNAs, which is supported by the observed upregulation of developmental myosin isoforms [49]. Furthermore, in the model proposed by Vogler et al., this elevated muscle regeneration would trigger the formation of sarcoplasmic myo-granules whose number, function and regulation could then be further disturbed by the aggregation-prone TDP-43^p.Trp385IlefsTer10^ resulting in autophagic myodegeneration over time [59].

Finally, this finding adds TDP-43 to the growing list of RNA-binding proteins as genetic causes of myopathies. Interestingly, similar to TDP-43, frameshift mutations in the PrLDs of HNRNPA2B1 and HNRNPA1 that change the prion-like behaviour of the mutant proteins have been described recently as the genetic cause of myopathies [6, 29], whereas most of the missense variants in the exact same protein domain can be linked to a broader clinical spectrum including neurodegeneration [7, 28].

Although work on TDP-43^p.Trp385IlefsTer10^ is in early stages, the findings we report here will help to understand a muscle-specific function for TDP-43 and how it can be related with the development of myopathies. Furthermore, our work points towards a broadening of the disease spectrum of TDP-43 proteinopathies, expanding from pure neurodegenerative to muscular disorders.

## Supplementary Material

1

## Figures and Tables

**Table 1:** Clinical characteristics of family members of the Belgian myopathy family

	Sex	Age	Onset	Signs and symptoms	EMG	Muscle imaging (MRI)	Nerve biopsy	Muscle biopsy
III.1	F	78	< 40	Progressive muscle weakness, lower limb paraesthesia, diagnosed as ‘hereditary motor and sensory neuropathy type 2’	NA	NA	NA	Rimmed vacuoles, suggestive for inclusion body myopathy
III.5	F	77	30	Slowly progressive symmetric proximal and distal muscle weakness of lower and later upper limbs, no sensory complaints, mildly elevated CK. Died age 78 due to respiratory insufficiency	Compatible with mild chronic axonal neuropathy with polyphasic MUAPs of increased magnitude	lower limbs: generalized muscle atrophy with fatty infiltration	Axonal degeneration of myelinated and non-myelinated nerves, degeneration of myelin sheaths	Hypertrophic fibres, internalized nuclei, atrophic fibres with multiple nuclei and debris; EM: lamellar deposits
IV.2	F	50	42	Slowly progressive muscle weakness in lower more than upper limbs, predominantly in proximal and distal lower limbs, myalgia, paraesthesia in feet, slightly elevated CK	Polyphasic MUAPs with low amplitudes, compatible with myopathy	muscle atrophy and signs of myositis (triceps surae)	NA	NA
IV.3	F	45	36	Progressive proximal more than distal muscle weakness in lower more than upper limbs, episodic painful muscle cramps, paraesthesia, slightly elevated CK	Normal at age 42	muscle atrophy with fatty infiltration in vastus lateralis and tensor fasciae latae, symmetrical	NA	NA
IV.4	F	50	37	Exercise intolerance, increased muscle fatigability, proximal more than distal muscle weakness in lower more than upper limbs, shortened Achilles tendons, absent tendon reflexes, myalgia, paraesthesia in lower limbs, moderately elevated CK	Polyphasic MUAP’s with low amplitudes and increased recruitment in most muscle, in some muscles slight chronic neurogenic pattern. Combined neurogenic and predominantly moderate spontaneous activity and myogenic involvement	fatty atrophy mainly of vastus lateralis and medialis, sartorius, tensor fasciae latae, adductor magnus and brevis, gastrocnemius and soleus, peroneal and anterior tibial muscles, distal flexor hallucis longus, all symmetrical	NA	Internalized nuclei, fibre splitting, atrophic fibres of each fibre type without grouping, rare necrotic fibres, vacuolization with large and small rimmed vacuoles. Many smaller fibres contain small p62 + inclusions (TDP43 not tested). Proliferation of dense and fatty connective tissue. No inflammatory changes
IV.5	M	45	38	Myalgia, proximal and distal muscle weakness of lower limbs, shortened Achilles tendons, mildly increased CK	Polyphasic MUAP’s with low amplitudes, slight spontaneous activity	NA	NA	Atrophic fibres of each fibre type, many containing small and large rimmed vacuoles; focal endomysial inflammation with CD3 + T cells and macrophages; fatty connective tissue replacement; many p62 + and TDP43 + inclusions
V.1	M	34	32	Muscle stiffness, slow recuperation after exercise, chronic migraine	Normal	NA	NA	NA
V.2	F	33	-	Asymptomatic	NA	NA	NA	NA
V.3	F	20	14	Exercise intolerance, exercise-induced muscle pain, mild proximal more than distal weakness, shortened Achilles tendons, normal CK	NA	NA	NA	NA

*NA* not available, *CK* creatine kinase, *MUAP* motor unit amplitude mean
