# Supplementary material for: C-terminal frameshift variant of TDP-43 with pronounced aggregation-propensity causes rimmed vacuole myopathy but not ALS/FTD
Source: Acta Neuropathol. 2023 Mar 31;145(6):793–814. doi: 10.1007/s00401-023-02565-1 (PMC10175433; doi:10.1007/s00401-023-02565-1)

C-terminal frameshift variant of TDP-43 with pronounced aggregation-propensity causes rimmed vacuole myopathy but not ALS/FTD

Pedro Ervilha Pereira[1,2*], Nika Schuermans[1,2*], Antoon Meylemans[3,4], Pontus LeBlanc[1,2], Lauren Versluys[1,2], Katie E. Copley[5,6], Jack D. Rubien[5], Christopher Altheimer[7], Myra Peetermans[1,2], Elke Debackere[1,2], Olivier Vanakker[1,2], Sandra Janssens[1,2], Jonathan Baets[8,9,10], Kristof Verhoeven[3,11], Martin Lammens[12], Sofie Symoens[1,2], Boel De Paepe[3,4], Sami J. Barmada[7], James Shorter[5,6], Jan L. De Bleecker[3,4], Elke Bogaert[1,2,#], Bart Dermaut[1,2,†,#]

Supplementary data

1. **Patient clinical characteristics**
2. **Supplementary tables**

1. Patient clinical characteristics

Patient III.1

This 78-year-old female patient was diagnosed at the age of 40 years with a molecularly unexplained hereditary motor and sensory neuropathy type 2 and consequently pronounced muscular atrophy. A muscle biopsy was performed, showing the presence of rimmed vacuoles which could be indicative of an inclusion body myopathy. She became wheelchair bound around the age of 60. Anamnestically, we learn that her father suffered from a very similar neurodegenerative disorder and died at the age of 50.

Patient III.4

This 77-year-old female patient presented with a progressive symmetric proximal muscle weakness of the lower and upper limbs, which was first noticed around the age of 30. She became wheelchair bound around the age of 50. Recently, a mild degree of spasticity was noticed by her treating neurologist. On multiple occasions, elevated CK-levels were found, ranging between 19-401 U/L. She had no sensory complaints. Electromyography at the time was most compatible with progressive neuropathy and secondary muscular atrophy. Nerve biopsy showed axonal degeneration of myelinated and non-myelinated nerves and secondary degeneration of the myelin sheaths. Muscle biopsy showed hypertrophic fibers with internalized nuclei and lamellar deposits and the presence of atrophic fibers with multiple nuclei and debris. Imaging showed generalized muscle atrophy with fatty infiltration of the lower and upper limbs, spinal and abdominal musculature.

Patient IV.2

This 50-year-old female patient consulted a neurologist at the age of 42 because of progressive muscle weakness, predominantly in the lower limbs. Additionally, she complained of myalgia and paresthesia in her legs. She had consistently elevated blood CK-levels ranging between 259-544 U/L. Ultrasound of the triceps surae performed for other reasons, showed severe muscle atrophy and signs of myositis. Electromyography showed polyphasic motor unit action potentials with low amplitudes, the signature of myopathic disease. No muscle biopsy was performed.

Patient IV.3

Patient IV.2 has a younger sister, now 45 years old, who first consulted a neurologist at the age of 36 because of progressive proximal muscle weakness. Furthermore, she complained of episodic painful cramping of the calf musculature and paresthesia in the lower limb. Multiple routine blood examinations revealed elevated CK levels up till 390 U/L. There were no abnormalities seen on electroneuromyography. MRI of the quadriceps showed muscle atrophy with pronounced fatty infiltration.

Patient IV.4

This 50-year-old female patient started to experience exercise intolerance and increased fatigability around the age of 37. Two years later, she was seen by a neurologist who objectified a proximal muscle weakness and noticed a positive Gower's sign and a steppage gait. She had shortened Achilles tendons and absent tendon reflexes on clinical examination. At that time, she complained of myalgia and paresthesia in the lower limbs. Over the years, she had consistently elevated CK-levels ranging from 672 to 1533 U/L. Electroneuromyography showed polyphasic motor unit action potentials with low amplitudes and low compound muscle action potential amplitude, reflecting a combination of myogenic and neurogenic disease. Lower limb MRI showed atrophy of proximal and distal leg musculature with fatty infiltration. The quadriceps and triceps surae were most severely affected, the adductor and popliteal muscles were relatively spared. Muscle biopsy showed internalized nuclei, fiber splitting, atrophic and necrotic fibers (predominantly type 1 muscle fibers), vacuolization with the presence of rimmed vacuoles, proliferation of connective tissue and fatty transformation. Immunohistochemistry of sarcolemmal membrane proteins was normal.

Patient IV.5

This patient is the 45-year-old younger brother of patient IV.4. At the age of 38 he consulted an orthopedic surgeon because of chronic pain in both legs, associated with difficulties to climb stairs. Because of the presence of quadriceps atrophy, shortened Achilles tendons and the inability to walk on his toes or heels, he was referred to a neurologist. The tentative diagnosis of a myopathy was confirmed by electromyography which showed polyphasic MUAP's with low amplitudes and decreased CMAP amplitudes. There were no sensory nerve conduction aberrations. CK-levels were consistently increased as high as 368 U/L. Muscle biopsy shows the presence of atrophic fibers with rimmed vacuoles indicative of a myopathy.

Patient V.1

This patient is the 35-year-old grandson of patient II.1. His mother died at the age of 38 due to liver disease. She had no neuromuscular problems at the time. Since the age of 32, he complained of muscle stiffness and slow muscle recuperation after exercise. The complaints are worsening over time. Additionally, he suffers from chronic migraine, for which he is treated with propranolol, valproate and topiramate. An EMG was performed and was normal.

Patient V.2

This patient is the 33-year-old younger sister of patient IV.1. At this point, she is asymptomatic.

Patient V.3

This is the 20-year-old daughter of patient IV.4, born at a gestational age of 39 weeks via an urgent Caesarean section because of intrauterine growth restriction and bradycardia. In the neonatal period she was diagnosed with growth hormone deficiency and hypothyroidism due to an ectopic posterior pituitary and hypotrophic anterior pituitary for which substitution therapy was initiated. At the age of 6, she developed mild unilateral hydronephrosis secondary to a mature cystic teratoma of the left ovary, which was resected shortly after. Upon physical examination mild dysmorphic features were noticed: small stature, trigonal face, sparse eyebrows, prominent ears with low implantation and normal rotation, prominent chin, relatively large forehead, and a high arched palate. A clear developmental delay was noticed. Around the age of 14 she started to complain of exercise intolerance and exercise-induced muscle pain. Mild muscle atrophy, absent lower limb reflexes and shortened Achilles tendons were seen during physical examination at that time. Additionally, a discrete positive Gower’s sign was present. Blood CK levels were normal. Polysomnography, performed at the age of 16, showed mild hypoventilation.

2. Supplementary tables

Supplementary Table 1. Rare variants identified in the 4.6 Mb region at 1p36.21-22 (LOD-score 3.61).

**Variant**	**Population Data**	**Patient Genotype/Phenotype**	**Databases**	**Predictive data**	**Functional data**	**Segregation data**	**Allelic data**
MFN2 (c.1403G>A, NM_014874.3)	-Present in GnomAD;<br>-A.D. or X-Linked;<br>-BS2: >=2 hemi-/homozygotes or 0.1% - 1%;	NA	-BP6: benign according to reliable databases;	NA	NA	-PP1: variant segregates with disease in multiple family members;<br>-PP1_PS: >4 family members;	NA
VPS13D (c.1299G>A, ENST00000011700)	-Present in GnomAD;<br>-A.R.;<br>-BS2: >=2 homozygotes or 0.3% - 2%;	NA	-BP6: benign according to reliable databases;	-BP4: predictions indicate variant is benign;<br>-BP4: missense, splice or intronic variant;	NA	-PP1: variant segregates with disease in multiple family members;<br>-PP1_PS: >4 family members;	NA
TARDBP (c.1152_1162del, NM_007375.3)	-PM2: Variant not in GnomAD	NA	NA	-PP3: predictions indicate variant is harmful;	NA	-PP1: variant segregates with the disease in multiple family members;	NA

				-PVS1: nonsense, frameshift, +/- 1 or +/- 2 splice site or exon deletion;		-PP1_PS: >4 family members;	
UBIAD1 (c.224C>T, NM_013319.2)	-Present in GnomAD;<br>-A.D. or X-Linked;<br>-BS2: >=2 hemi-/homozygotes or 0.1% - 1%;	NA	-BP5: benign according to reliable databases;	-BP4: predictions indicate variant is benign;<br>-BP4: missense, splice or intronic variant;	NA	-PP1: variant segregates with disease in multiple family members;<br>-PP1_PS: >4 family members;	NA

Supplementary table 2. List of *Drosophila* lines used.

**Line Number/Name**	**Line genotype**	**Origin**	**Reference**
**Driver lines**			
Tub-Gal80	y w; tub-gal80ts; tubulin-gal4/TM6B	**Flystock P.Callaerts**	**-**
D42-Gal4	w[*]; P{w[+mW.hs]=GawB}D42	BDSC	BDSC 8816
nSyb-Gal4	y[1] w[*]; P{w[+m*]=nSyb-GAL4.S}3	BDSC	BDSC 51635
Tub-Gal80ts	w[*]; P{w[+mC]=tubP-GAL80[ts]}20; TM2/TM6B, Tb[1]	BDSC	BDSC 7019
nSyb-Gal4 (TM6B)	IF/CyO; nSyb-Gal4/TM6B	Generated in-house	-
Mef2-Gal4	IF/CyO; Mef2-Gal4/TM3	**Flystock P.Dourlen**	BDSC27390
Mef2-Gal80	tub-Gal80ts/CyO; Mef2-Gal4/TM6B, Tb	Generated in-house	-
nSyb-Gal80	tub-Gal80ts/CyO; nSyb-Gal4/TM6B, Tb	Generated in-house	-
**UAS-TARDBP transgene lines**			
UAS-TDP43 WT	pUAST TDP wild type AttP2 m4m1/TM3	Genetivision	-
UAS-TDP43(A382T)	pUAST TDP p.Ala382Thr AttP2 m1m1/TM3	Genetivision	-
UAS-TDP43(M337V)	pUAST TDP p.Met337Val AttP2 m1m1/TM3	Genetivision	-
UAS-TDP43(11bp)	pUAST TDP Trp385IlefsTer10 AttP2 m2m1/TM3	Genetivision	-
**TBPH deletion lines**			
TBPHΔ23M9	TBPHΔ23m9/CyO:GFP	Gift from David Morton	-

Table S3. Median survival of fly lines overexpressing the TDP-43 constructs in study (nsyb-GAL4 driver). P-value relative to control lines, * p≤0.05, ** p≤0.01, *** p≤0.001, **** p<=0.0001. Statistical analysis performed with a Kaplan-Meir test.P-value relative to control lines, **** p<=0.0001. Statistical analysis performed with a non-parametric Krustal-Wallis test.

	Fly line	***Median survival (days)***	***Significance***
Male flies	*Luciferase-Control*	*70*	
	TDP-43 WT	*7*	****
	TDP-43^{A382T}	*7*	****
	TDP-43^{M337V}	*5*	****
	TDP-43^{Trp385IlefsTer10}	*79*	****
Female flies	*Luciferase-Control*	*70*	
	TDP-43 WT	*3*	****
	TDP-43^{A382T}	*4*	****
	TDP-43^{M337V}	*4*	****
	TDP-43^{Trp385IlefsTer10}	*91*	****

Table S4. Median survival of fly lines overexpressing the TDP-43 constructs (adult-onset expression, different drivers). P-value relative to control lines, * p≤0.05, ** p≤0.01, *** p≤0.001, **** p<=0.0001. Statistical analysis performed with a Kaplan-Meir test.

	Fly line	***Median survival (days)***	***Significance***
Tub-Gal4 driver	*Luciferase-Control*	*46*	
	TDP-43 WT	*14*	****
	TDP-43^{A382T}	*14*	****
	TDP-43^{Trp385IlefsTer10}	*42*	**
nSyb-Gal80 driver	*Luciferase-Control*	*46*	
	TDP-43 WT	*14*	****
	TDP-43^{A382T}	*16*	****
	TDP-43^{Trp385IlefsTer10}	*44*	****
Mef2-Gal4 driver	*Luciferase-Control*	*46*	
	TDP-43 WT	*30*	****
	TDP-43^{A382T}	*32*	****
	TDP-43^{Trp385IlefsTer10}	*49*	***n.s.***

Table S5. List of antibodies used.

**Primary antibodies**	**Brand**	**Reference number**
TDP-43 (N-terminal)	ProteinTech	10782-2-AP
pTDP-43 [pS409/410]	CosmoBio	CAC-TIP-PTD-M01
pTDP-43 [pS369]	Courtesy of Prof. E. Buratti	-
GAPDH	Invitrogen	MA5-15738-1MG
β-Tubulin	Invitrogen	32-2600
β-Actin (HRP-conjugated)	Santa Cruz Biotechnology	sc-47778 HRP
**Secondary Antibodies**	**Brand**	**Reference number**
Anti-Rabbit AlexaFluor 488	Invitrogen	A21206
Anti-Mouse AlexaFluor 555	Invitrogen	A31570
Anti-Rabbit IgG HRP-linked	Invitrogen	G-21234
Anti-Mouse IgG HRP-linked	Invitrogen	G-21040

References

1. Dobin, A. *et al.* STAR: ultrafast universal RNA-seq aligner. *Bioinformatics* **29**, 15–21 (2013).

2. Li, H. *et al.* The Sequence Alignment/Map format and SAMtools. *Bioinformatics* **25**, 2078–2079 (2009).

3. Hartley, S. W. & Mullikin, J. C. QoRTs: a comprehensive toolset for quality control and data processing of RNA-Seq experiments. *BMC Bioinformatics* **16**, 224 (2015).

4. Robinson, J. T. *et al.* Integrative genomics viewer. *Nature biotechnology* vol. 29 24–26 (2011).

5. Robinson, M. D., McCarthy, D. J. & Smyth, G. K. edgeR: a Bioconductor package for differential expression analysis of digital gene expression data. *Bioinformatics* **26**, 139–140 (2010).

6. Subramanian, A. *et al.* Gene set enrichment analysis: a knowledge-based approach for interpreting genome-wide expression profiles. *Proc. Natl. Acad. Sci. U. S. A.* **102**, 15545–15550 (2005).

7. Mootha, V. K. *et al.* PGC-1alpha-responsive genes involved in oxidative phosphorylation are coordinately downregulated in human diabetes. *Nat. Genet.* **34**, 267–273 (2003).

8. Zhou, Y. *et al.* Metascape provides a biologist-oriented resource for the analysis of systems-level datasets. *Nat. Commun.* **10**, 1523 (2019).

9. Shen, S. *et al.* rMATS: robust and flexible detection of differential alternative splicing from replicate RNA-Seq data. *Proc. Natl. Acad. Sci. U. S. A.* **111**, E5593-601 (2014).

Suppl figure 1

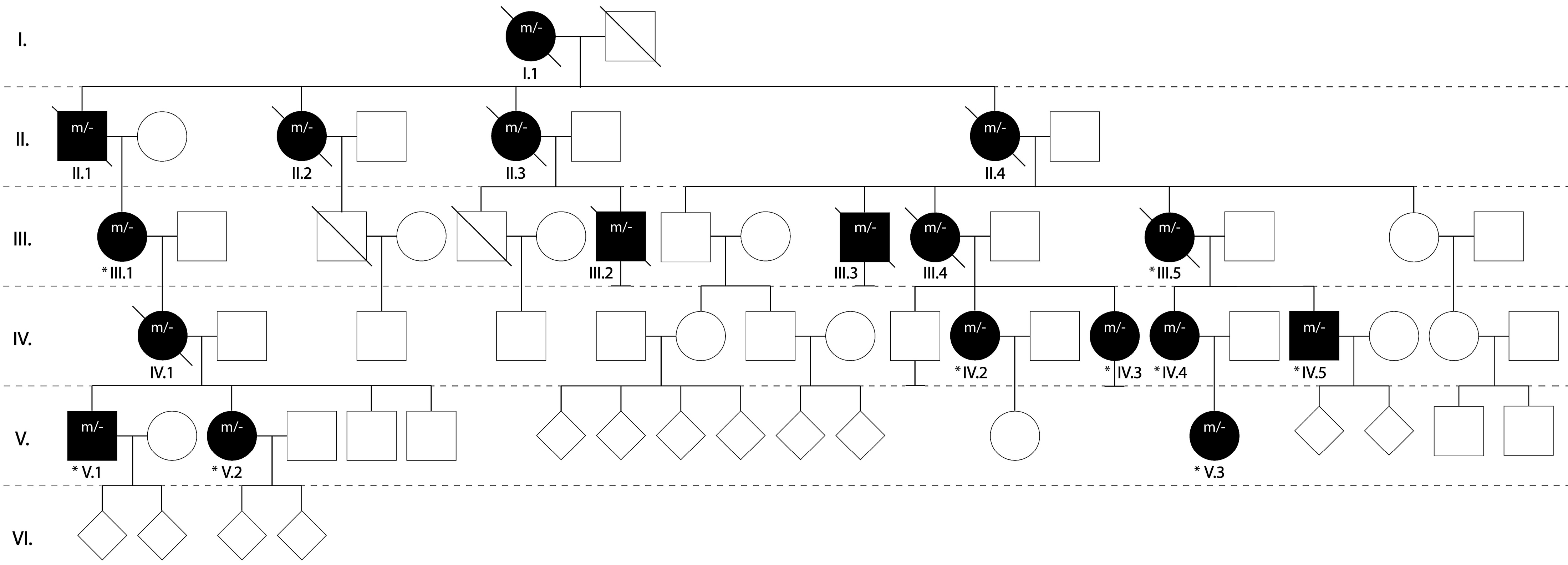

Suppl figure 2

a

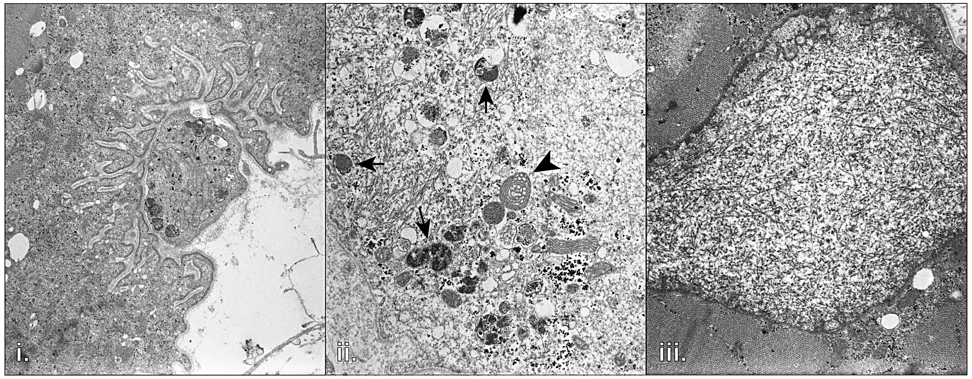

b

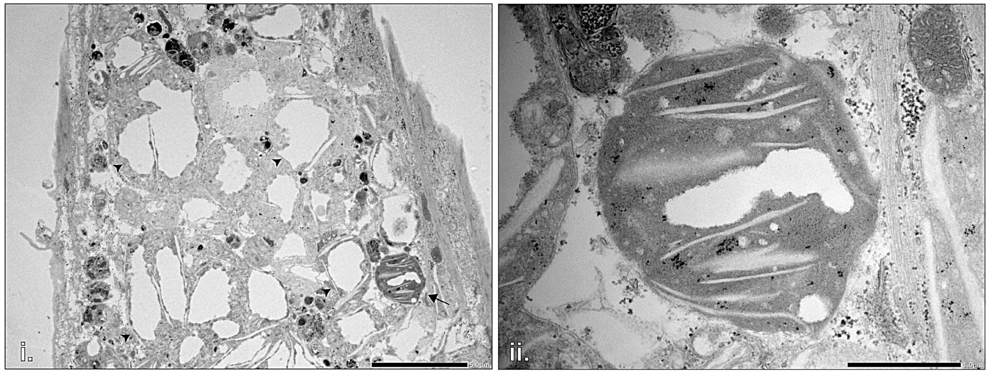

c

Marker Name	Marker Position	Affected																										Un-Affected																				Unknown			
		Typed																		Un-Typed								Typed		Un-Typed																		Un-Typed			
		III.1		III.4		IV.2		IV.3		IV.4		IV.5		V.1		V.2		V.3		II.1		II.4		III.3		IV.1		Daughter of IV.2		Spouse of II.1		Spouse of II.4		Spouse of III.1		Spouse of III.3		Spouse of III.4		Spouse of IV.1		Spouse of IV.2		Spouse of IV.4		I.1		Spouse of I.1	
rs11121117	6.9834	1	5	7	1	11	1	11	1	13	1	13	1	15	9	16	1	19	13	1	3	1	3	7	1	9	1	17	11	5	6	7	8	9	10	11	12	13	14	15	16	17	18	19	20	1	2	3	4
rs299699	7.1222	1	5	7	1	11	1	11	1	13	1	13	1	15	9	16	1	19	13	1	3	1	3	7	1	9	1	17	11	5	6	7	8	9	10	11	12	13	14	15	16	17	18	19	20	1	2	3	4
rs12410893	7.6449	1	5	7	1	11	1	11	1	13	1	13	1	15	9	16	1	19	13	1	3	1	3	7	1	9	1	17	11	5	6	7	8	9	10	11	12	13	14	15	16	17	18	19	20	1	2	3	4
rs875148	7.8151	1	5	7	1	11	1	11	1	13	1	13	1	15	9	16	1	19	13	1	3	1	3	7	1	9	1	17	11	5	6	7	8	9	10	11	12	13	14	15	16	17	18	19	20	1	2	3	4
rs797263	8.0046	1	5	7	1	11	1	11	1	13	1	13	1	15	9	16	1	19	13	1	3	1	3	7	1	9	1	17	11	5	6	7	8	9	10	11	12	13	14	15	16	17	18	19	20	1	2	3	4
rs3753163	8.0845	1	5	7	1	11	1	11	1	13	1	13	1	15	9	16	1	19	13	1	3	1	3	7	1	9	1	17	11	5	6	7	8	9	10	11	12	13	14	15	16	17	18	19	20	1	2	3	4
rs4908842	8.1534	1	5	7	1	11	1	11	1	13	1	13	1	15	9	16	1	19	13	1	3	1	3	7	1	9	1	17	11	5	6	7	8	9	10	11	12	13	14	15	16	17	18	19	20	1	2	3	4
rs10864439	8.6690	1	5	7	1	11	1	11	1	13	1	13	1	15	1	16	1	19	1	1	3	1	3	7	1	9	1	17	11	5	6	7	8	9	10	11	12	13	14	15	16	17	18	19	20	1	2	3	4
rs12144133	8.9702	1	5	7	1	11	1	11	1	13	1	13	1	15	1	16	1	19	1	1	3	1	3	7	1	9	1	17	11	5	6	7	8	9	10	11	12	13	14	15	16	17	18	19	20	1	2	3	4
rs17396382	9.0563	1	5	7	1	11	1	11	1	13	1	13	1	15	1	16	1	19	1	1	3	1	3	7	1	9	1	17	11	5	6	7	8	9	10	11	12	13	14	15	16	17	18	19	20	1	2	3	4
rs912963	9.1141	1	5	7	1	11	1	11	1	13	1	13	1	15	1	16	1	19	1	1	3	1	3	7	1	9	1	17	11	5	6	7	8	9	10	11	12	13	14	15	16	17	18	19	20	1	2	3	4
rs2182326	9.1517	1	5	7	1	11	1	11	1	13	1	13	1	15	1	16	1	19	1	1	3	1	3	7	1	9	1	17	11	5	6	7	8	9	10	11	12	13	14	15	16	17	18	19	20	1	2	3	4
rs2506878	9.3322	1	5	7	1	11	1	11	1	13	1	13	1	15	1	16	1	19	1	1	3	1	3	7	1	9	1	17	11	5	6	7	8	9	10	11	12	13	14	15	16	17	18	19	20	1	2	3	4
rs556506	9.5067	1	5	7	1	11	1	11	1	13	1	13	1	15	1	16	1	19	1	1	3	1	3	7	1	9	1	17	11	5	6	7	8	9	10	11	12	13	14	15	16	17	18	19	20	1	2	3	4
rs11121675	9.8261	1	5	7	1	11	1	11	1	13	1	13	1	15	1	16	1	19	1	1	3	1	3	7	1	9	1	17	11	5	6	7	8	9	10	11	12	13	14	15	16	17	18	19	20	1	2	3	4
rs1033638	9.8528	1	5	7	1	11	1	11	1	13	1	13	1	15	1	16	1	19	1	1	3	1	3	7	1	9	1	17	11	5	6	7	8	9	10	11	12	13	14	15	16	17	18	19	20	1	2	3	4
rs4845853	10.0095	1	5	7	1	11	1	11	1	13	1	13	1	15	1	16	1	19	1	1	3	1	3	7	1	9	1	17	11	5	6	7	8	9	10	11	12	13	14	15	16	17	18	19	20	1	2	3	4
rs2982384	10.1678	1	5	7	1	11	1	11	1	13	1	13	1	15	1	16	1	19	1	1	3	1	3	7	1	9	1	17	11	5	6	7	8	9	10	11	12	13	14	15	16	17	18	19	20	1	2	3	4
rs2922240	10.2599	1	5	7	1	11	1	11	1	13	1	13	1	15	1	16	1	19	1	1	3	1	3	7	1	9	1	17	11	5	6	7	8	9	10	11	12	13	14	15	16	17	18	19	20	1	2	3	4
rs2745285	10.3929	1	5	7	1	11	1	11	1	13	1	13	1	15	1	16	1	19	1	1	3	1	3	7	1	9	1	17	11	5	6	7	8	9	10	11	12	13	14	15	16	17	18	19	20	1	2	3	4
rs4491070	10.8961	1	5	7	1	11	1	11	1	13	1	13	1	15	1	16	1	19	1	1	3	1	3	7	1	9	1	17	11	5	6	7	8	9	10	11	12	13	14	15	16	17	18	19	20	1	2	3	4
rs6690493	10.9016	1	5	7	1	11	1	11	1	13	1	13	1	15	1	16	1	19	1	1	3	1	3	7	1	9	1	17	11	5	6	7	8	9	10	11	12	13	14	15	16	17	18	19	20	1	2	3	4
rs653667	11.0179	1	5	7	1	11	1	11	1	13	1	13	1	15	1	16	1	19	1	1	3	1	3	7	1	9	1	17	11	5	6	7	8	9	10	11	12	13	14	15	16	17	18	19	20	1	2	3	4
rs235256	11.0623	1	5	7	1	11	1	11	1	13	1	13	1	15	1	16	1	19	1	1	3	1	3	7	1	9	1	17	11	5	6	7	8	9	10	11	12	13	14	15	16	17	18	19	20	1	2	3	4
rs2493875	11.4769	1	5	7	1	11	1	11	1	13	1	13	1	15	1	16	1	19	1	1	3	1	3	7	1	9	1	17	11	5	6	7	8	9	10	11	12	13	14	15	16	17	18	19	20	1	2	3	4
rs3000861	11.5419	1	5	7	1	11	1	11	1	13	1	13	1	15	1	16	1	19	1	1	3	1	3	7	1	9	1	17	11	5	6	7	8	9	10	11	12	13	14	15	16	17	18	19	20	1	2	3	4
rs3010876	11.5456	1	5	7	1	11	1	11	1	13	1	13	1	15	1	16	1	19	1	1	3	1	3	7	1	9	1	17	11	5	6	7	8	9	10	11	12	13	14	15	16	17	18	19	20	1	2	3	4
rs12135103	11.6819	1	5	7	1	11	1	11	1	13	1	13	1	15	1	16	1	19	1	1	3	1	3	7	1	9	1	17	11	5	6	7	8	9	10	11	12	13	14	15	16	17	18	19	20	1	2	3	4
rs4345799	12.6171	1	5	7	1	11	1	11	1	13	1	13	1	15	1	16	1	19	1	1	3	1	3	7	1	9	1	17	11	5	6	7	8	9	10	11	12	13	14	15	16	17	18	19	20	1	2	3	4
rs2245218	12.9059	1	5	7	1	11	1	11	1	13	1	13	1	15	1	16	1	19	1	1	3	1	3	7	1	9	1	17	11	5	6	7	8	9	10	11	12	13	14	15	16	17	18	19	20	1	2	3	4
rs3820012	12.9162	1	5	7	1	11	1	11	1	13	1	13	1	15	1	16	1	19	1	1	3	1	3	7	1	9	1	17	11	5	6	7	8	9	10	11	12	13	14	15	16	17	18	19	20	1	2	3	4
rs1203626	12.9437	1	5	7	1	11	1	11	1	13	1	13	1	15	1	16	1	19	1	1	3	1	3	7	1	9	1	17	11	5	6	7	8	9	10	11	12	13	14	15	16	17	18	19	20	1	2	3	4
rs9287293	12.9746	1	5	7	1	11	1	11	1	13	1	13	1	15	1	16	1	19	1	1	3	1	3	7	1	9	1	17	11	5	6	7	8	9	10	11	12	13	14	15	16	17	18	19	20	1	2	3	4
rs10737914	12.9768	1	5	7	1	11	1	11	1	13	1	13	1	15	1	16	1	19	1	1	3	1	3	7	1	9	1	17	11	5	6	7	8	9	10	11	12	13	14	15	16	17	18	19	20	1	2	3	4
rs1528787	12.9804	1	5	7	1	11	1	11	1	13	1	13	1	15	1	16	1	19	1	1	3	1	3	7	1	9	1	17	11	5	6	7	8	9	10	11	12	13	14	15	16	17	18	19	20	1	2	3	4
rs657359	13.2797	1	5	7	1	11	1	11	1	13	1	13	1	15	1	16	1	19	1	1	3	1	3	7	1	9	1	17	11	5	6	7	8	9	10	11	12	13	14	15	16	17	18	19	20	1	2	3	4
rs12406699	13.3641	1	5	7	1	11	1	11	1	13	1	13	1	15	1	16	1	19	1	1	3	1	3	7	1	9	1	17	11	5	6	7	8	9	10	11	12	13	14	15	16	17	18	19	20	1	2	3	4
rs12129808	13.4548	1	5	7	1	11	1	11	1	13	1	13	1	15	1	16	1	19	1	1	3	1	3	7	1	9	1	17	11	5	6	7	8	9	10	11	12	13	14	15	16	17	18	19	20	1	2	3	4
rs10803278	13.5388	1	5	7	1	11	1	11	1	13	1	13	1	15	1	16	1	19	1	1	3	1	3	7	1	9	1	17	11	5	6	7	8	9	10	11	12	13	14	15	16	17	18	19	20	1	2	3	4
rs12564154	13.5556	1	5	7	1	11	1	11	1	13	1	13	1	15	1	16	1	19	1	1	3	1	3	7	1	9	1	17	11	5	6	7	8	9	10	11	12	13	14	15	16	17	18	19	20	1	2	3	4
rs12733612	13.5921	1	5	7	1	11	1	11	1	13	1	13	1	15	1	16	1	19	1	1	3	1	3	7	1	9	1	17	11	5	6	7	8	9	10	11	12	13	14	15	16	17	18	19	20	1	2	3	4
rs10927466	13.6561	1	5	7	1	11	1	11	1	13	1	13	1	15	1	16	1	19	1	1	3	1	3	7	1	9	1	17	11	5	6	7	8	9	10	11	12	13	14	15	16	17	18	19	20	1	2	3	4
rs3765380	13.9563	1	5	7	1	11	3	11	3	13	1	13	1	15	1	16	1	19	1	1	3	1	3	7	3	9	1	17	11	5	6	7	8	9	10	11	12	13	14	15	16	17	18	19	20	1	2	3	4
rs2807555	13.9921	1	5	7	1	11	3	11	3	13	1	13	1	15	1	16	1	19	1	1	3	1	3	7	3	9	1	17	11	5	6	7	8	9	10	11	12	13	14	15	16	17	18	19	20	1	2	3	4
rs761288	14.1437	1	5	7	1	11	7	11	3	13	1	13	1	15	1	16	1	19	1	1	3	1	3	7	3	9	1	17	11	5	6	7	8	9	10	11	12	13	14	15	16	17	18	19	20	1	2	3	4
rs3752934	14.1908	1	5	7	1	11	7	11	3	13	1	13	1	15	1	15	1	19	1	1	3	1	3	7	3	9	1	17	11	5	6	7	8	9	10	11	12	13	14	15	16	17	18	19	20	1	2	3	4
rs2235788	14.2089	1	5	7	1	11	7	11	3	13	1	13	1	15	1	15	1	19	1	1	3	1	3	7	3	9	1	17	11	5	6	7	8	9	10	11	12	13	14	15	16	17	18	19	20	1	2	3	4
rs4661582	14.2490	1	5	7	1	11	7	11	3	13	1	13	1	15	1	15	1	19	1	1	3	1	3	7	3	9	1	17	11	5	6	7	8	9	10	11	12	13	14	15	16	17	18	19	20	1	2	3	4
rs742362	14.4928	1	5	7	1	11	7	11	3	13	1	13	1	15	1	15	1	19	1	1	3	1	3	7	3	9	1	17	11	5	6	7	8	9	10	11	12	13	14	15	16	17	18	19	20	1	2	3	4

Suppl figure3

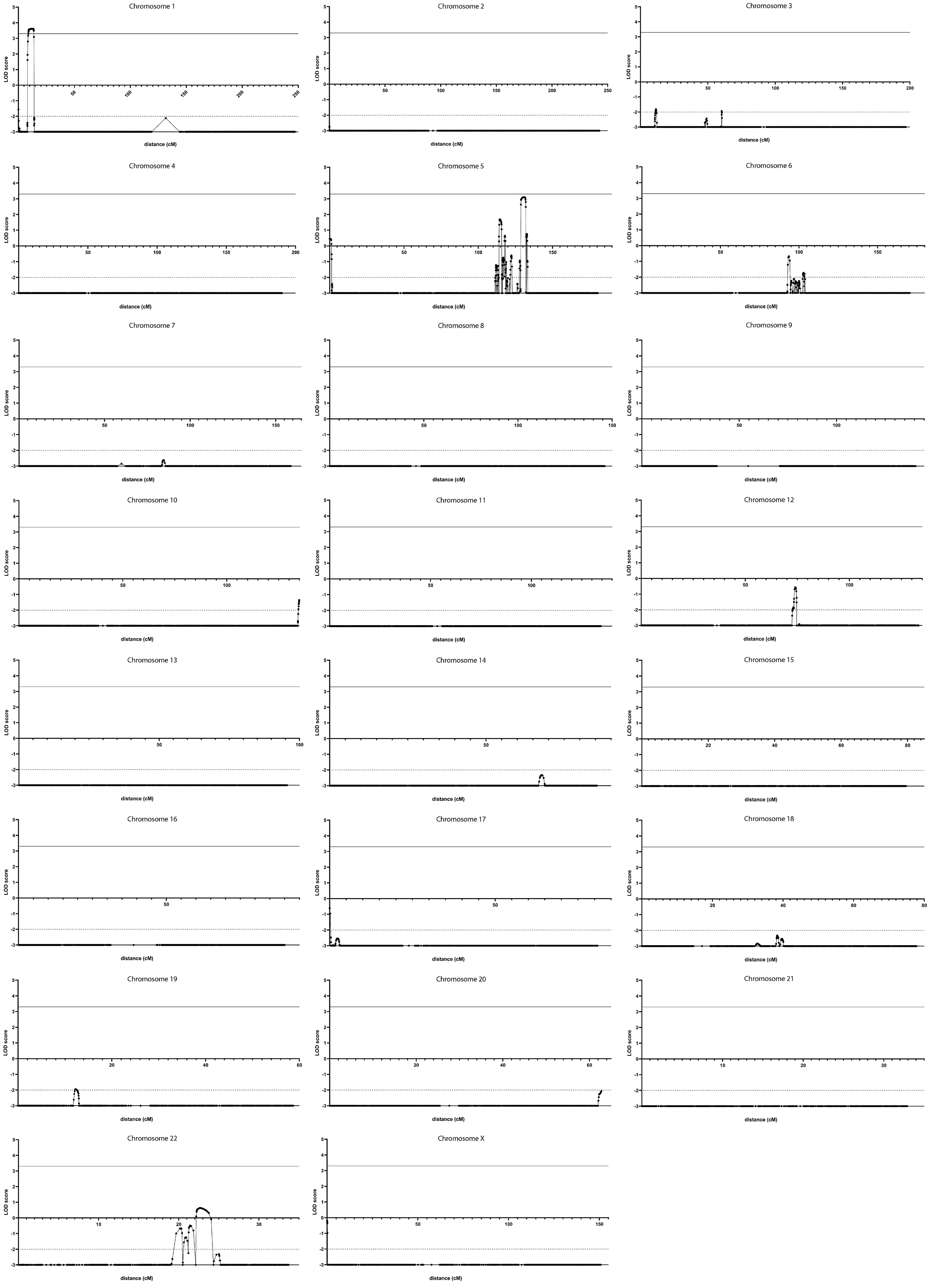

Suppl figure 4

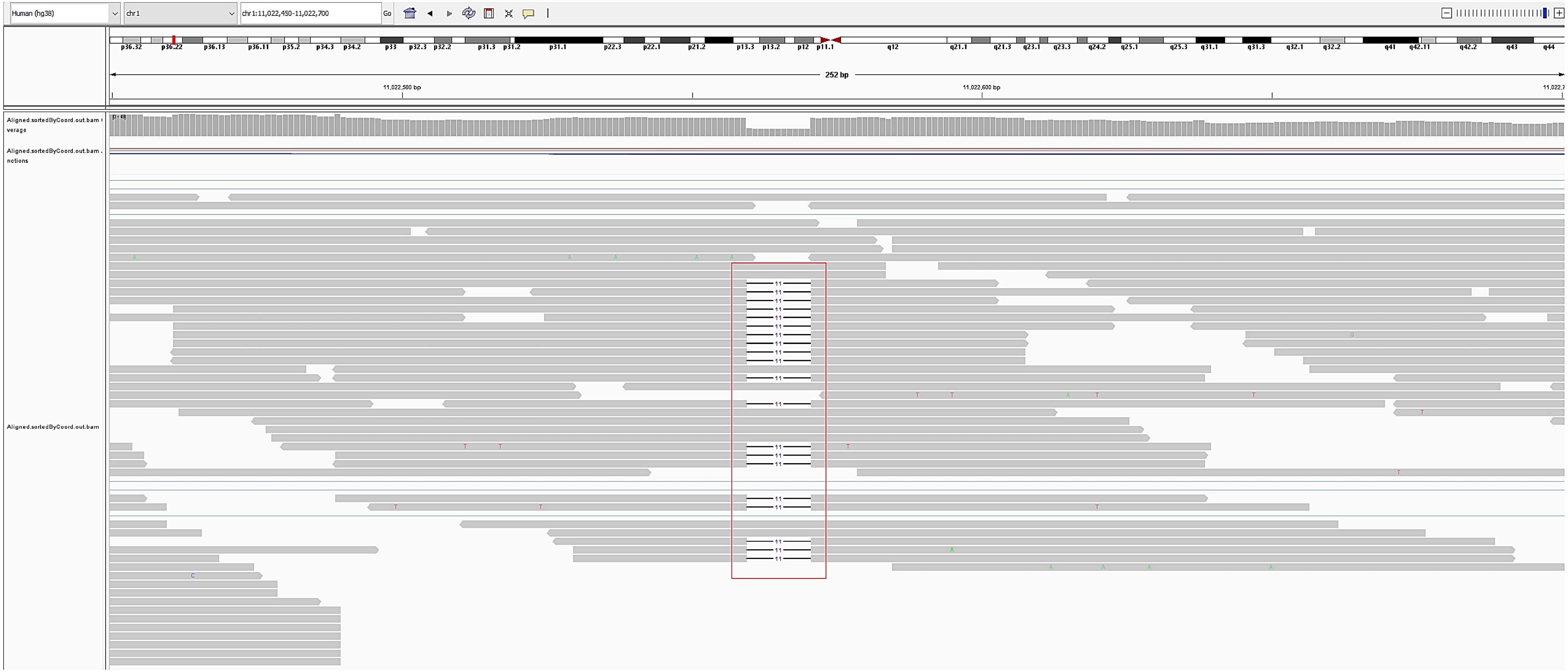

Suppl figure 5

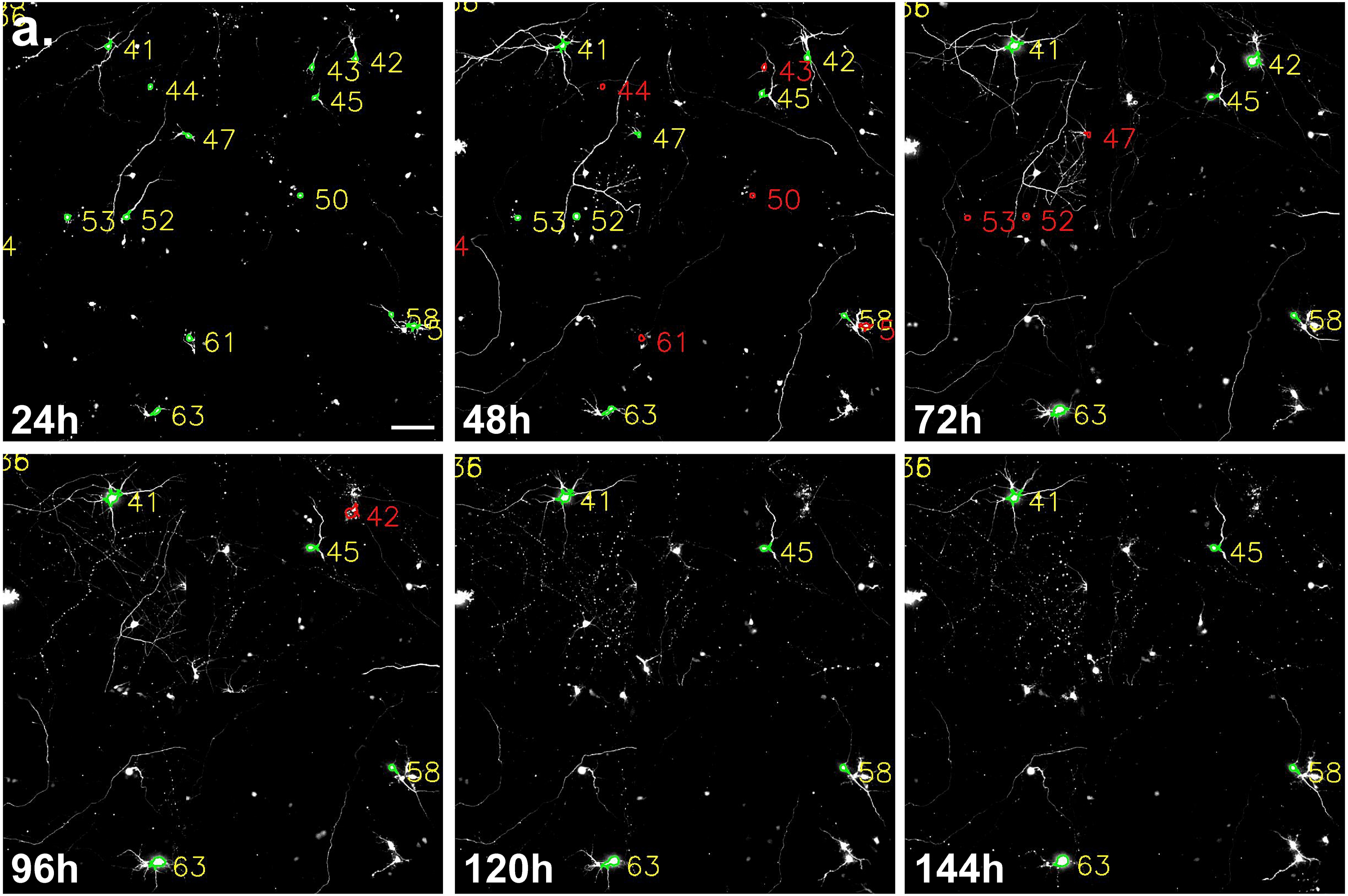

Supplement: Supplementary file 1 [file 401_2023_2565_MOESM1_ESM.pdf]
